# An RNA thermometer in the chloroplast genome of *Chlamydomonas* facilitates temperature-controlled gene expression

**DOI:** 10.1093/nar/gkad816

**Published:** 2023-10-19

**Authors:** Kin Pan Chung, F Vanessa Loiacono, Juliane Neupert, Mengting Wu, Ralph Bock

**Affiliations:** Max-Planck-Institut für Molekulare Pflanzenphysiologie, Department Organelle Biology, Biotechnology and Molecular Ecophysiology, Am Mühlenberg 1, D-14476 Potsdam-Golm, Germany; Max-Planck-Institut für Molekulare Pflanzenphysiologie, Department Organelle Biology, Biotechnology and Molecular Ecophysiology, Am Mühlenberg 1, D-14476 Potsdam-Golm, Germany; Max-Planck-Institut für Molekulare Pflanzenphysiologie, Department Organelle Biology, Biotechnology and Molecular Ecophysiology, Am Mühlenberg 1, D-14476 Potsdam-Golm, Germany; Max-Planck-Institut für Molekulare Pflanzenphysiologie, Department Organelle Biology, Biotechnology and Molecular Ecophysiology, Am Mühlenberg 1, D-14476 Potsdam-Golm, Germany; Max-Planck-Institut für Molekulare Pflanzenphysiologie, Department Organelle Biology, Biotechnology and Molecular Ecophysiology, Am Mühlenberg 1, D-14476 Potsdam-Golm, Germany

## Abstract

Riboregulators such as riboswitches and RNA thermometers provide simple, protein-independent tools to control gene expression at the post-transcriptional level. In bacteria, RNA thermometers regulate protein synthesis in response to temperature shifts. Thermometers outside of the bacterial world are rare, and in organellar genomes, no RNA thermometers have been identified to date. Here we report the discovery of an RNA thermometer in a chloroplast gene of the unicellular green alga *Chlamydomonas reinhardtii*. The thermometer, residing in the 5′ untranslated region of the *psaA* messenger RNA forms a hairpin-type secondary structure that masks the Shine–Dalgarno sequence at 25°C. At 40°C, melting of the secondary structure increases accessibility of the Shine–Dalgarno sequence to initiating ribosomes, thus enhancing protein synthesis. By targeted nucleotide substitutions and transfer of the thermometer into *Escherichia coli*, we show that the secondary structure is necessary and sufficient to confer the thermometer properties. We also demonstrate that the thermometer provides a valuable tool for inducible transgene expression from the *Chlamydomonas* plastid genome, in that a simple temperature shift of the algal culture can greatly increase recombinant protein yields.

## Graphical Abstract

**Graphical Abstract:**
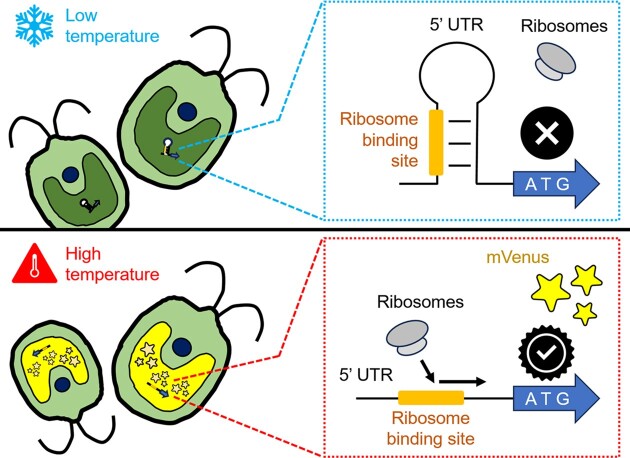

## Introduction

Inducibility of gene expression is usually conferred by multi-component systems that are composed of a *cis*-acting element at the DNA or RNA level, and a *trans*-acting regulator protein and/or a small molecule (metabolite) as inducer. RNA thermometers differ from this general concept in that they function independently of *trans*-acting inducers. Two types of RNA thermometers have been described: zipper-like and switch-like (1). Both types of RNA thermometer consist of temperature-sensitive messenger RNA (mRNA) secondary structures in the 5′ untranslated region (5′ UTR), which harbour the ribosome-binding site, also known as Shine–Dalgarno sequence (2). RNA thermometers are believed to function according to a simple thermodynamic principle. Examples of the zipper-like RNA thermometers are the ROSE (repression of heat shock gene expression) elements (3,4). At low growth temperatures, the 5′ UTR is structured via complementary base pairing and, consequently, the ribosome-binding site is masked (i.e. trapped in a double-stranded conformation). As the Shine–Dalgarno sequence needs to be single-stranded to be accessible by the 30S ribosomal subunit for translation initiation, protein synthesis is largely repressed at low growth temperatures. On shifting to higher growth temperatures, the secondary structure melts, the ribosome-binding site becomes exposed and translation initiation can occur at higher rates. By contrast, switch-like RNA thermometers allow translation at low temperature. A well-studied example is the *cspA*, which is responsible for the cold shock response (5). At high temperature, the *cspA* mRNA forms a secondary structure involving an interaction of the 5′ UTR with the coding region. The interaction masks the ribosome-binding site, thereby suppressing translation. When the temperature drops, the *cspA* mRNA undergoes a conformational change that exposes the ribosome-binding site. As a result, the switch between these alternate and mutually exclusive conformations dictates the accessibility of the Shine–Dalgarno sequence.

Natural RNA thermometers have been mainly found in bacteria. In pathogenic bacteria, they can be involved in the expression of virulence genes in response to temperature changes in the host during the infection process (6–9). The secondary structures of natural RNA thermometers are often complex, and so are the conformational changes that the RNA structure undergoes upon temperature changes (10–12). To identify potential RNA thermometers, a bioinformatic platform that focuses on the prediction of RNA structural properties has been established (13). However, relatively simple stem-loop-type structures have also been found to act as thermometers (12). In addition, minimal-size RNA thermometers have been designed synthetically. By adjusting the free energy of stem-loop structures to the physiological temperature range and testing various relevant parameters (e.g. stem size and sequence, loop size), it has been possible to construct small synthetic thermometers that confer temperature responsiveness to bacterial translation (14–16). Recently, using thermodynamic computations, synthetic RNA thermometers with diverse temperature responses (differing in sensitivity and threshold) have been developed (17). Synthetic RNA thermometers have also become important parts and attractive modules in synthetic biology, as they are compatible with a wide range of expression hosts, and their function neither requires *trans*-acting protein factors nor chemical inducers (18). While providing valuable RNA-only tools to biotechnology and synthetic biology, due to their mode of action, both natural and synthetic thermometers usually display a certain leakiness and thus, cannot serve as tight on/off switches of gene expression. Nevertheless, RNA thermometers have been used to produce recombinant proteins in various bacteria, including *Escherichia coli*, *Yersinia pseudotuberculosis* and *Pseudomonas putida* (14,19,20).

The small genome of the chloroplast is of bacterial origin, and the basic mechanisms of translation have been retained over more than a billion years of evolution (21,22). This high degree of conservation includes the use of Shine–Dalgarno sequences for translation initiation of many (but not all) chloroplast genes (23–25). Although no RNA thermometers have been found in any chloroplast (or mitochondrial) genome, in view of the retention of Shine–Dalgarno-dependent translation initiation, it seems conceivable that RNA thermometers could function also in gene expression of the chloroplast. This appears to be an attractive possibility, because tools for inducible gene and transgene expression in engineered organellar genomes are scarce. The few available tools often rely on additional nuclear transgenes (26–31), which, however, are undesirable, because they abrogate one of the key advantages of organellar engineering: the increased transgene containment due to the maternal mode of organelle inheritance (32). Notable exceptions are a Lac repressor-based expression system (33), a riboswitch-based induction system in tobacco plastids (34–36) and a cold-inducible system based on translational read-through in the chloroplast of *Chlamydomonas* (37). Each of these systems has its advantages and disadvantages, with the latter including dependence on external application of (often toxic) inducer molecules and/or substantial leakiness under non-inducing conditions.

To explore the potential of RNA thermometers for the control of plastid gene expression, here we have tested synthetic RNA thermometers in the chloroplast genome of the model alga *Chlamydomonas reinhardtii*. We find that previously reported thermometers (designed for bacteria) do not permit gene expression in the chloroplast at physiological temperatures. Surprisingly, we discovered an endogenous element in the 5′ UTR of the chloroplast *psaA* mRNA that acts as an RNA thermometer and confers temperature-dependent expression onto transgenes in both chloroplasts and bacteria.

## Materials and methods

### Algal strains and growth conditions

*Chlamydomonas reinhardtii* strain TN72 (Δ*psbH*, cell wall deficient) was used as recipient strain for chloroplast transformation (38). The strain was maintained on agar-solidified Tris-acetate-phosphate (TAP) medium at 22–25°C under continuous low light (10 μE m^−2^ s^−1^), or in liquid TAP medium on a rotary shaker (120 rpm) at 25°C under continuous low light (10 μE m^−2^ s^−1^). Chloroplast-transformed (transplastomic) lines were selected and maintained on high-salt minimal (HSM) medium (39). The cell density was measured by cell counting using a Z2 Coulter Counter (Beckman Coulter, Krefeld, Germany). For heat induction experiments, cultures were grown in liquid TAP medium (15 ml) at 25°C to stationary phase, then transferred to two Erlenmeyer flasks (5 ml each) and incubated at 40°C for 6 h (with shaking at 120 rpm and continuous illumination at 100 μE m^−2^ s^−1^). To record temperature response curves (by conducting growth experiments at 20, 25, 30, 35 and 40°C), cultures were grown in liquid TAP medium (30 ml) at 20°C to stationary phase, then transferred to new Erlenmeyer flasks (5 ml each) and incubated at the designated temperatures for 6 h (with shaking at 120 rpm and continuous illumination at 100 μE m^−2^ s^−1^).

### Bacterial strains and growth conditions

*Escherichia coli* strain One Shot™ TOP10 (Invitrogen, Karlsruhe, Germany) was used as recipient strain for bacterial transformation experiments. Heat-shock transformation was performed and the resultant transformants were selected on agar-solidified Luria–Bertani (LB) medium with ampicillin (100 μg/ml) at 37°C. To study RNA thermometer function, temperature-dependent induction of gene expression was assayed. To this end, individual colonies were picked and inoculated in liquid LB medium (with 100 μg/ml ampicillin) at 18°C on a rotary shaker (180 rpm). When the optical density at 600 nm (OD_600_) of the cultures had reached 0.6, they were diluted with fresh LB medium and grown at the designated temperatures (18, 25, 37 or 42°C) until their OD_600_ reached 1.0. Samples were then taken for protein isolation. To study the temperature effect on p24-Nef accumulation, individual colonies of the corresponding transformed strain (40) were picked and inoculated in liquid LB medium (with 100 μg/ml ampicillin) at 25°C on a rotary shaker (180 rpm). When the OD_600_ of the cultures had reached 0.6, they were diluted with fresh LB medium and grown at 25 or 40°C until their OD_600_ reached 1.0. Samples were then taken for protein isolation.

### Vector construction

The mVenus-HA, HMGB1-FLAG, Cpl-1-HA and p24-Nef coding sequences were codon-optimized for expression in *Chlamydomonas* chloroplasts using the Kazusa CAI table (41), and chemically synthesized (GeneArt, Regensburg, Germany). Transformation plasmids were generated by inserting the corresponding transgene into the chloroplast expression vectors pASapI (*atpA* promoter and 5′ UTR) and pSRSapI (*psaA* promoter and 5′ UTR) using the SapI and SphI restriction sites (38). Chimeric expression elements combining the *atpA* promoter with the *psaA* 5′ UTR (PatpA:psaA), the *psaA* promoter with the *atpA* 5′ UTR (PpsaA:atpA), the *psaA* promoter with the SRT_U0 5′ UTR and the *psaA* promoter with the SRT_U6 5′ UTR (14) were produced by PCR and inserted into the corresponding expression vectors using the unique MluI and NcoI restriction sites. Green fluorescent protein (GFP) expression vectors for *E. coli* were generated by inserting versions of the *psaA* 5′ UTR (WT, ΔHP and ΔSD) between the rRNA operon promoter and the GFP coding sequence using the BamHI and NcoI restriction sites. mVenus-HA expression vectors with mutated hairpin structures (m1–m4) were generated by inserting modified versions of the *psaA* 5′ UTR between the *psaA* promoter and the mVenus-HA coding sequence, using the HiFi DNA assembly cloning kit (New England Biolabs). PCR amplification was performed with Phusion High-Fidelity DNA Polymerase (Thermo Fisher) following the manufacturer's instructions. All primers used in this study are listed in Supplementary Table S1. The cloning of all vectors was done in *E. coli* strain DH5α. Large scale plasmid preparations were performed using the NucleoBond Xtra Midi kit (Macherey-Nagel).

### Chloroplast transformation of *Chlamydomonas reinhardtii*

For chloroplast transformation experiments, the glass bead transformation method was applied (42). Cultures of the TN72 strain were grown to early logarithmic phase (i.e. a cell density of 1 × 10^6^ cells/ml) in liquid TAP medium. Cells were then harvested by centrifugation at 3000*g* for 5 min, and the cell pellet was resuspended in TAP medium to reach a cell density of 1 × 10^8^ cells/ml. Samples of 300 μl of the cell suspension were mixed with 0.3 g glass beads (Sigma-Aldrich) and 5 μg circular plasmid DNA of the transformation vector. The mixture was then vortexed for 15 s at maximum speed, followed by spreading on HSM plates. The plates were incubated in a growth chamber at 22–25°C under continuous light (100 μE m^−2^ s^−1^) for 4 weeks to select for photoautotrophy.

### Fluorescence measurements with the microplate reader

Candidate transformed colonies were picked from selection plates and incubated in 100 μl of TAP medium in transparent 96-well plates (Corning™ Costar™ 96-well flat-bottom microplates; Thermo Fisher). Cells were grown to mid-logarithmic phase in a growth chamber, followed by fluorescence measurement using the CLARIOstar® microplate reader (BMG LABTECH GmbH, Ortenberg, Germany). Excitation at 494 nm (bandwidth 18 nm) and emission at 538 nm (bandwidth 20 nm) were used to measure mVenus fluorescence intensity. The OD_750_ was determined for normalization. Background subtraction and data analysis was performed by the MARS Data Analysis Software (BMG LABTECH GmbH, Ortenberg, Germany).

### Protein isolation

Total protein extracts of *Chlamydomonas* were prepared for SDS–PAGE and western blot analyses. To this end, algal cells from 3-ml cultures were harvested by centrifugation at 3000*g* for 5 min, and the cell pellet was resuspended in 200 μl of protein extraction buffer [50 mM HEPES/KOH pH 7.5, 10 mM KAc, 5 mM MgAc, 1 mM EDTA, 1% Triton X-100, 1 mM DTT, 1× protease inhibitor cocktail cOmplete (Roche)]. Cell lysis was induced by repeated resuspension (15 times) using a 27G needle with a 1-ml syringe. The resulting cell lysate was incubated on ice for 30 min, followed by centrifugation at 15 000*g* for 15 min. The supernatant was recovered as protein extract and denatured with SDS–PAGE sample buffer at 95°C for 5 min. Total protein extracts of *E. coli* were prepared as follows. Cultures were grown at the designated temperatures until the OD_600_ had reached 1.0, then 3-ml aliquots of the cultures were harvested by centrifugation at 5000*g* for 5 min. The cell pellets were frozen in liquid nitrogen and resuspended in 400 μl of lysis buffer (50 mM HEPES, 300 mM NaCl, 0.5% SDS, pH 8.0). The samples were then incubated on ice for 30 min, followed by sonication (amplitude 10%, 15 s; Sonifier, W-250 D, G. Heinemann Ultraschall und Labortechnik, Germany). Subsequently, the lysates were centrifuged at 5000*g* for 10 min, the supernatants were recovered as protein extracts and denatured in SDS–PAGE sample buffer at 95°C for 5 min.

### SDS–PAGE and western blot analysis

Protein samples were electrophoretically separated in denaturing 12% SDS-PAA gels, followed by transfer onto PVDF (polyvinylidene difluoride) membranes (HybondTM; GE Healthcare, UK) using standard blotting techniques. The membranes were then incubated with blocking buffer (5% milk powder in 1× TBS-T) at room temperature for 1 h. After several washing steps with TBS-T, the membranes were incubated with the primary antibody at 4°C overnight using the following antibody dilutions: anti-GFP (JL-8; Clontech, 1:1000), anti-FLAG (F7425, Sigma-Aldrich, 1:1000), anti-HA (THE^TM^ HA Tag, GenScript, 1:1000), anti-p24 (ab9071, Abcam, 1:1000) and anti-PsaA (AS06172, Agrisera, 1:1000). Subsequently, an HRP-conjugated secondary antibody (mouse, AS111772, 1:25 000; rabbit, AS09602, 1:10 000, Agrisera) was added and the membranes were incubated at room temperature for 1 h. The ECL Plus^TM^ detection system was used for chemiluminescence signal detection. Staining of the membranes with Ponceau S or staining of the gel with Coomassie Brilliant Blue was performed as control for equal loading. Recombinant GFP (rGFP; Roche) was used as standard. Relative protein abundance was quantified by band intensity measurement using the Fiji software (https://imagej.net/software/fiji/). Statistical analysis was performed using Welch's *t*-test.

### Confocal laser-scanning microscopy

Confocal microscopy suing a TCS SP8 instrument (Leica) was performed to study the chloroplast expression of mVenus-HA. mVenus fluorescence was detected using an argon laser for excitation (at 514 nm), and recording the fluorescence emission at 525–555 nm. Chlorophyll fluorescence was detected using an argon laser for excitation (at 514 nm), and analysing emission at 650–700 nm. For GFP detection in *E. coli*, excitation with an argon laser at 488 nm was used and emission was analysed at 500–545 nm.

### RNA secondary structure prediction

The RNA secondary structures of the synthetic thermometers SRT_U0 and SRT_U6 were adopted from a previously published study (14). Prediction of the RNA secondary structures of the *atpA* and *psaA* 5′ UTRs was performed using the algorithms of the Mfold web server (v.2.3; 43) and the ViennaRNA RNAfold web server (44).

### RNA isolation and northern blot analyses

Total RNA was extracted from algal cultures incubated at the designated temperatures (20, 25, 30 or 40°C) using the TRIzol^®^ reagent (Thermo Fisher Scientific) following the protocol of the manufacturer. The RNA yield was determined with the Nanodrop instrument (Thermo Fisher Scientific). *mVenus-HA* and *psaA* expression was assessed by northern blot analyses performed as described previously (45). A radioactively labelled RNA probe corresponding to the coding sequence of mVenus was synthesized with the MAXIscript^®^ Kit (Thermo Fisher Scientific) following the manufacturer's protocol and using [α-32P]-UTP (Hartmann Analytic GmbH). The template was PCR-amplified using primers oVL528 and oVL529 listed in Supplementary Table S1. A hybridization probe for exon 3 of *psaA* was generated by PCR amplification using primers oKPC326 and oKPC327 (Supplementary Table S1), and radiolabelled by random priming with [α-32P]-dCTP.

## Results

### Temperature inducibility of gene expression from the 5′ UTR of the plastid *psaA* gene in algal plastids

**Figure 1.**
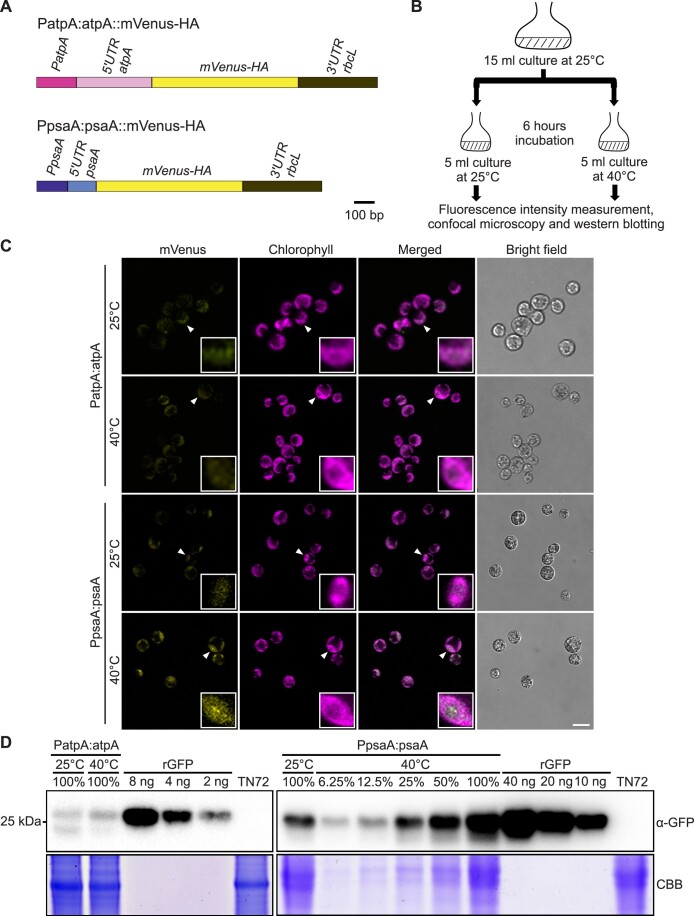
Identification of a putative RNA thermometer for temperature-controlled transgene expression in *Chlamydomonas reinhardtii* chloroplasts. **(A)** Maps of mVenus-HA expression cassettes introduced into the chloroplast genome of *Chlamydomonas*. mVenus-HA constructs driven by the *psaA* promoter and 5′ UTR (PpsaA:psaA) and the *atpA* promoter and 5′ UTR (PatpA:atpA) were introduced into the chloroplast genome by stable transformation. **(B)** Workflow to determine mVenus-HA expression levels and test for RNA thermometer activity. **(C)** Confocal laser-scanning microscopy to visualize the expression of mVenus-HA in the chloroplast of the transplastomic strains at 25 and 40°C. Enlarged insets (right bottom corner) show the chloroplast localization of the mVenus fluorescence signal in individual cells (marked by arrowheads). Scale bar, 10 μm. **(D)** Immunoblot analysis of the thermoinducibility of reporter protein expression. On heat induction, total protein samples were extracted from cultures incubated at 25 and 40°C. For immunodetection of the reporter protein mVenus-HA, an anti-GFP antibody (α-GFP) was used (mVenus is a GFP variant, and therefore, can be detected with anti-GFP antibodies). The lower bands (<25 kDa) represent a degradation product of mVenus-HA (that lacks the C-terminal HA-tag). Samples of 20 μg protein were loaded as 100%. A dilution series of recombinant green fluorescent protein (rGFP) was used as standard. The sample (PpsaA:psaA, 40°C) was loaded in a 2-fold dilution series (from 100% to 6.25%) to provide a semiquantitative assessment of protein abundance. Protein extract of the untransformed strain TN72 was used as a negative control. Coomassie Brilliant Blue (CBB) staining of the protein gel was performed to provide a loading control.

We previously developed a set of small synthetic RNA thermometers that comprise a simple stem-loop structure in the 5′ UTR harbouring the Shine–Dalgarno sequence (14). The thermometers conferred temperature inducibility of gene expression in *Escherichia coli* by melting of the RNA secondary structure at elevated temperatures. To test whether such synthetic RNA thermometers (SRTs) can also be used to modify gene expression in chloroplasts in a temperature-dependent manner, we introduced synthetic thermometers (SRT_U0 and SRT_U6; 14) fused to a reporter gene encoding an HA-tagged version of the mVenus fluorescent protein into the chloroplast genome of the green alga *Chlamydomonas reinhardtii* (Supplementary Figures S1 and S2). Two control constructs were included in our initial tests, in which reporter expression was driven by (i) the *psaA* promoter and 5′ UTR, and (ii) the *atpA* promoter and 5′ UTR, both from *Chlamydomonas* plastids (Figure 1A). These expression elements (PatpA:atpA and PpsaA:psaA) are frequently used to drive transgene expression in *Chlamydomonas* chloroplasts (46,47).

Chloroplast transformation was performed using the established glass bead transformation protocol (42). Transformed (transplastomic) algal clones were selected by restoration of photoautotrophic growth in a non-photosynthetic recipient strain harbouring a disrupted gene for the essential photosystem II subunit PsbH in its chloroplast genome (38,42) (Supplementary Figure S1). Homoplasmic transplastomic strains were isolated (42) and tested for thermoinducibility by comparing mVenus fluorescence intensities at two growth temperatures (25 vs 40°C; Figure 1B). The synthetic thermometers (SRT_U0 and SRT_U6) showed no expression of the reporter (Supplementary Figure S2C,D), possibly due to the lack of stabilizing element(s) at the 5′ end of the synthetic 5′ UTR, which makes the chimeric mRNA prone to degradation in *Chlamydomonas* chloroplasts (48).

Surprisingly, we did not observe the expected constitutive, temperature-independent expression from the *psaA* promoter and 5′ UTR. Instead, we noticed a strong increase in reporter protein accumulation and mVenus fluorescence in the PpsaA:psaA transplastomic strain, when the algal cultures were shifted from 25 to 40°C (Figure 1C,D). By contrast, the *atpA* expression elements were not responsive to temperature, and both mVenus fluorescence (Figure 1C) and protein accumulation (Figure 1D) were very similar at 25 and 40°C. This finding suggests that thermoinducibility is not a general property of chloroplast gene expression in *Chlamydomonas*, but may be a specific property of the *psaA* promoter and/or 5′ UTR.

**Figure 2.**
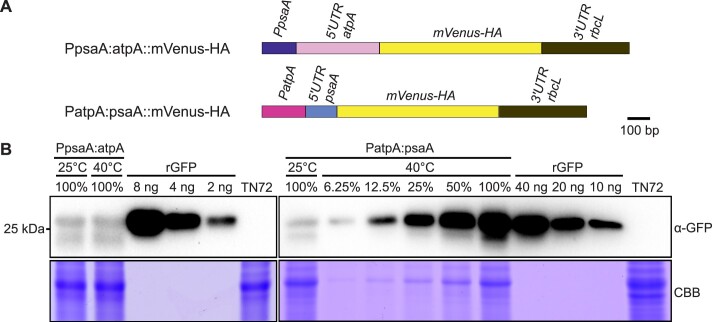
The *psaA* 5′ UTR confers temperature inducibility of reporter protein expression in *Chlamydomonas* chloroplasts. **(A)** Maps of mVenus-HA expression cassettes driven by the *psaA* promoter and the *atpA* 5′ UTR (PpsaA:atpA), or the *atpA* promoter in combination with the *psaA* 5′UTR (PatpA:psaA). **(B)** Immunoblot analysis of the thermoinducibility of reporter protein accumulation. On heat induction, total protein samples were extracted from cultures incubated at 25 or 40°C. Western blotting was performed with an anti-GFP antibody (α-GFP) to determine the abundance of mVenus-HA. Samples of 20 μg protein were loaded as 100%. A dilution series of recombinant GFP (rGFP) was used as standard. The sample (PatpA:psaA, 40°C) was loaded in a 2-fold dilution series (from 100% to 6.25%) to provide a semiquantitative assessment of protein abundance. Protein extract of the untransformed strain TN72 was used as a negative control. Coomassie Brilliant Blue (CBB) staining of the protein gel was performed to provide a loading control.

Next, we wanted to examine whether the temperature inducibility of reporter protein expression from the *psaA* expression elements comes from the promoter or the 5′ UTR. To this end, we designed two new chloroplast transformation constructs, in which we (i) exchanged the *psaA* 5′ UTR by the *atpA* 5′UTR (PpsaA:atpA::mVenus-HA; Figure 2A), and (ii) replaced the *psaA* promoter by the *atpA* promoter (PatpA:psaA::mVenus-HA; Figure 2A). While combination of the *psaA* promoter with the *atpA* 5′ UTR showed no temperature inducibility, strong induction of mVenus-HA expression was seen in the strain expressing the reporter from the *atpA* promoter and the *psaA* 5′ UTR (Figure 2B). These observations strongly suggest that the thermoinducibility of reporter protein expression is conferred by the *psaA* 5′ UTR.

### Identification of a putative RNA thermometer in the *psaA* 5′ UTR

Having shown that the *psaA* 5′ UTR confers thermoinducibility whereas the *atpA* 5′ UTR does not, we next sought to identify the structural basis of the temperature-dependent expression from the *psaA* 5′ UTR. Since all known RNA thermometers function by temperature-induced conformational changes in the RNA secondary structure, we conducted an *in silico* analysis of the secondary structures of the mRNA sequences encompassing the 5′ UTR and the start codon region for both *psaA* and *atpA*. As expected, both UTRs could be readily folded into complex secondary structures (Supplementary Figures S3 and S4), consistent with a large body of previous work that has implicated structured 5′ UTR sequences in translational regulation and the control of mRNA stability in chloroplasts (49,50). Interestingly, whereas the *atpA* sequence showed no secondary structure in the region immediately upstream of the AUG start codon (Supplementary Figure S4), the *psaA* 5′ UTR exhibited a stem-loop structure upstream of the start codon (Supplementary Figure S3). Notably, this structure contains the Shine–Dalgarno sequence and is reminiscent of the secondary structures reported for previously designed minimal-size synthetic RNA thermometers (14). This finding raises the possibility that, by shifting the growth temperature from 25 to 40°C, the Shine–Dalgarno sequence-containing stem-loop structure is melted, thus unmasking the ribosome-binding site and facilitating efficient translation initiation.

### The *psaA* stem-loop structure functions as RNA thermometer in *E. coli*

**Figure 3.**
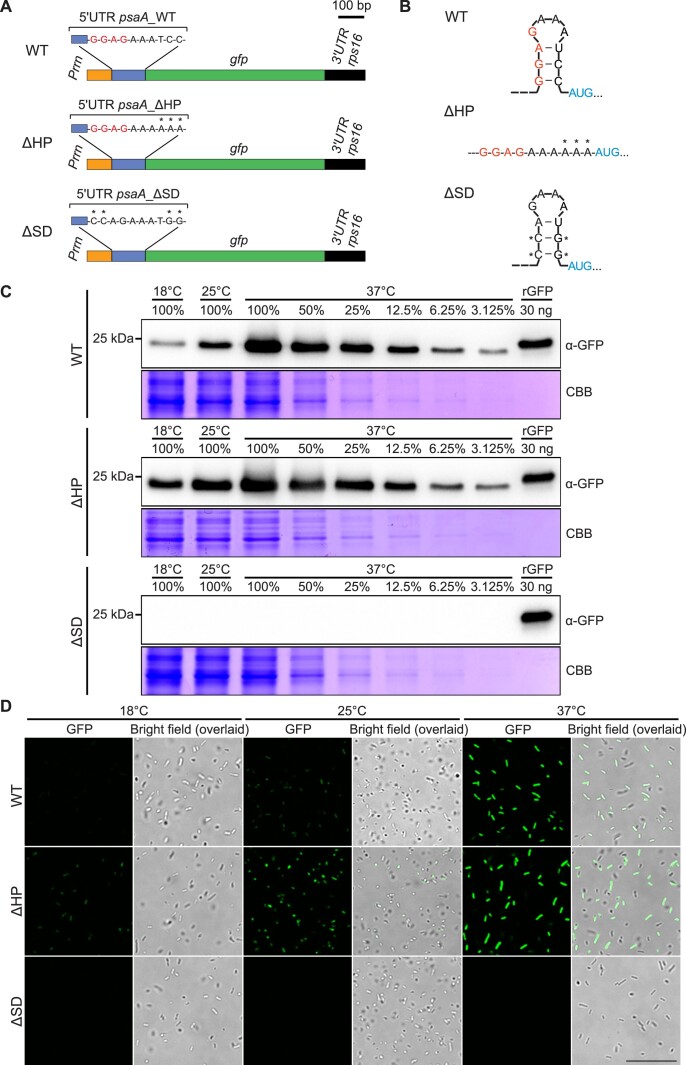
The *psaA* 5′ UTR functions as an RNA thermometer in *E. coli*. **(A)** Schematic maps of GFP expression cassettes driven by the rRNA operon promoter (Prrn) and different versions of the chloroplast *psaA* 5′ UTR from *Chlamydomonas* (WT, ΔHP and ΔSD). The Shine–Dalgarno sequence is indicated in red. Mutated nucleotides are denoted with asterisks. HP, hairpin. SD, Shine–Dalgarno sequence. **(B)** Predicted RNA secondary structures of the WT, ΔHP and ΔSD *psaA* 5′ UTR sequences. The Shine–Dalgarno sequence is marked in red and the translation initiation codon in blue. Introduced nucleotide substitutions are indicated by asterisks. **(C)** Temperature-dependent accumulation of the reporter protein GFP. On heat induction, total protein was extracted from bacterial cultures incubated at 18, 25 or 37°C. Immunoblot detection was performed with an anti-GFP antibody (α-GFP). The 2-fold dilution series of the samples (WT, 37°C) and (ΔHP, 37°C) were loaded to provide a semiquantitative assessment of protein abundance. Coomassie Brilliant Blue (CBB) staining of the protein gel was performed to provide a loading control. **(D)** Visualization of temperature-dependent GFP accumulation in *E. coli* by confocal laser-scanning microscopy. GFP fluorescence was assayed in transformed bacterial cultures incubated at 18, 25 and 37°C, respectively. Scale bar, 10 μm.

The identification of a putative RNA thermometer in the *psaA* 5′ UTR and its potentially small size and simple structure prompted us to investigate whether the observed temperature responsiveness of gene expression was independent of *trans*-acting protein factors present in the chloroplast. We, therefore, set out to test whether the small stem-loop structure is necessary and sufficient to obtain temperature responsiveness of reporter gene expression in *E. coli*. To this end, the putative *psaA* thermometer structure was integrated into a previously designed reporter construct (using GFP as the reporter) suitable to test 5′ UTRs for temperature-responsive translation in *E. coli* (14). To examine whether the RNA secondary structure is required for temperature responsiveness, the hairpin was removed by introducing three nucleotide substitutions that eliminate base pairing in the stem structure (ΔHP construct; Figure 3A,B). As an additional control construct, four mutations were introduced that eliminate the Shine–Dalgarno sequence but retain the stem-loop structure (ΔSD construct; Figure 3A,B).

Comparison of reporter protein accumulation at three different temperatures (18, 25 and 37°C) in heat induction experiments revealed that the wild-type hairpin from the *psaA* 5′UTR conferred pronounced temperature responsiveness of gene expression in *E. coli*. By contrast, the effect of temperature on transgene expression is much less pronounced in the bacterial strain harbouring the ΔHP construct upon temperature shifts (Figure 3C). The remaining increase in protein accumulation with temperature probably reflects the general temperature dependence of the protein synthesis process (having its optimum at 37°C; 51) and the higher copy number of plasmids at elevated temperature (52). As expected, the two nucleotide substitutions in the Shine–Dalgarno sequence resulted in complete loss of reporter protein accumulation, suggesting that translation initiation in the *psaA* 5′ UTR is Shine–Dalgarno-dependent (Figure 3C).

To further confirm the function of the *psaA* hairpin as RNA thermometer in *E. coli*, GFP fluorescence in bacteria was analysed at the three growth temperatures by confocal laser-scanning microscopy. While the bacteria harbouring the ΔHP construct were highly fluorescent already when the culture was shifted to 25°C, the bacteria containing the wild-type *psaA* hairpin sequence showed strong fluorescence only when the incubation temperature was raised to 37°C (Figure 3D).

Taken together, the temperature responsiveness of the chloroplast *psaA* 5′ UTR in *E. coli* and its dependence on the hairpin structure strongly suggest that this element represents a genuine RNA thermometer.

### Modifications of the secondary structure alter thermometer properties in chloroplasts

**Figure 4.**
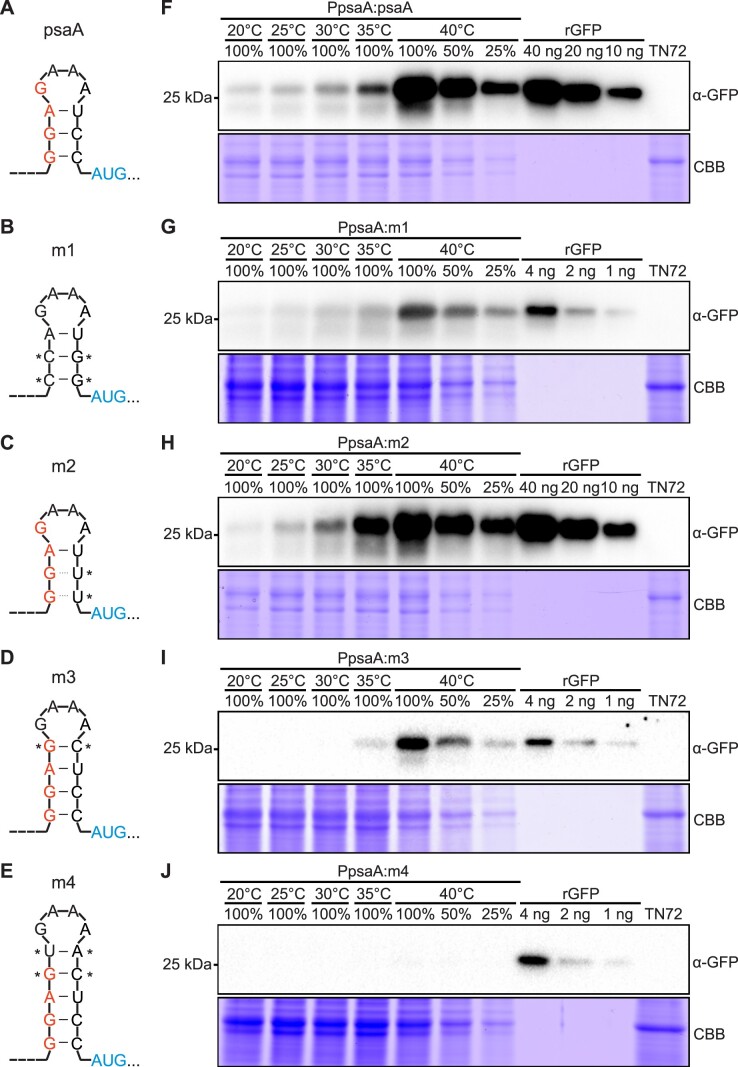
Modifications of the hairpin structure alter the thermometer properties of the *psaA* 5′ UTR. **(A-E)** RNA secondary structures of the hairpin in the native *psaA* 5′UTR and the modified hairpins m1, m2, m3 and m4. The Shine–Dalgarno sequence is indicated in red, the start codon site is shown in blue and mutated nucleotides are denoted with asterisks. In m1, the introduced point mutations result in loss of the Shine–Dalgarno sequence, but retention of the hairpin structure. By contrast, in variants m2 to m4, the stability of the hairpin structure is altered. The point mutations introduced in m2 weaken the hairpin structure, while insertion of additional base pairs in m3 and m4 create more stable hairpin structures. **(F–J)** Temperature-dependent reporter protein accumulation in chloroplasts of different transplastomic strains as assessed by immunoblotting. To determine the temperature responsiveness of different *psaA* 5′UTR variants, transplastomic strains were first grown at 20°C, then transferred to fresh medium (see Methods) and incubated at the designated temperatures (20, 25, 30, 35 and 40°C) for 6 hours. Total protein was extracted from the algal cultures, and accumulation of the reporter protein mVenus-HA was determined with an anti-GFP antibody (α-GFP). The 2-fold dilution series of the 40°C samples were loaded to provide a semiquantitative assessment of protein abundance. Samples of 20 μg protein were loaded as 100% in **(F)** and **(H)** and samples of 50 μg were loaded as 100% in **(G)**, **(I)** and **(J)**. A dilution series of recombinant GFP (rGFP) was used as standard. Protein extract of the untransformed strain TN72 was used as a negative control. Coomassie Brilliant Blue (CBB) staining of the protein gel was performed to provide a loading control.

If the hairpin structure within the *psaA* 5′ UTR is responsible for the RNA thermometer properties, it should be possible to modify the switch temperature by simply altering the stability (i.e. the melting temperature) of the structure (16). To test this idea, a series of mutated versions of the hairpin structure were constructed (Figure 4). mVenus-HA constructs driven by the *psaA* promoter and different versions of the *psaA* 5′UTR (psaA, m1, m2, m3 and m4) were introduced into the *Chlamydomonas* chloroplast genome by stable transformation (Figure 4A–E). The resulting transplastomic strains were then analysed by recording temperature response curves.

To confirm that the primary sequence of the hairpin is not required for temperature inducibility, two base pairs were flipped (variant m1; Figure 4B). These mutations alter the sequence of the hairpin, but fully retain the wild-type thermodynamic stability. Unavoidably, the two flipped base pairs eliminate the Shine–Dalgarno sequence, which results in strongly decreased translation rates, as expected (23). Nonetheless, temperature inducibility was retained (Figure 4F,G), consistent with temperature responsiveness being conferred by the secondary structure rather than the primary structure of the hairpin. When the stem of the hairpin was weakened by changing two GC base pairs to GU base pairs (variant m2; Figure 4C), substantial accumulation of the reporter protein was already observed at 30°C (Figure 4H), suggesting better accessibility of the Shine–Dalgarno sequence, presumably due to increased single-strandedness at 30°C. By contrast, when the stem of the hairpin was extended by one (variant m3; Figure 4D) or two (variant m4; Figure 4E) additional base pairs, reporter protein expression was undetectably low at temperatures between 20°C and 30°C (Figure 4I,J). In construct m4 with two extra base pairs, the response to temperature elevation was completely abolished (Figure 4J). Taken together, these results suggest that the strong secondary structure in variant m4 remains stable at elevated temperatures (and is not meltable at 40°C). Considering the increased switch temperature of the m3 and m4 thermometers, we attempted to test their thermoinducibility by incubating cultures at even higher growth temperatures. However, heat-induced bleaching and cell death were observed in algal cultures incubated at temperatures above 40°C (Supplementary Figure S5; 53), thus precluding the test of the more stable structures in m3 and m4 at higher growth temperatures in algal cells. As an alternative, we studied the thermoinducibility of the m3 and m4 variants at higher growth temperatures in bacteria. To this end, *E. coli* cells were transformed with constructs m3, m4 and psaA (as a control), and expression of the mVenus-HA reporter was determined at 37 and 42°C. mVenus-HA was detected in both m3 and m4 transformants at 42°C (Supplementary Figure S6), suggesting that these strong thermometer structures can be melted by further increasing the growth temperature.

### Temperature-dependent protein accumulation does not correlate with *mVenus-HA* mRNA levels

**Figure 5.**
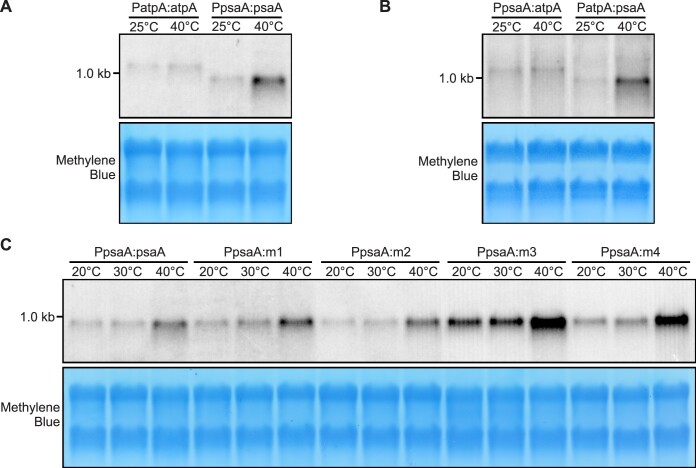
Expression analysis of the *mVenus-HA* transgene at the mRNA level in transplastomic algal strains upon heat induction. **(A)** Abundance of the *mVenus-HA* transcript in the PatpA:atpA and PpsaA:psaA transplastomic strains incubated at 25 and 40°C, respectively, was determined by northern blotting: 4 μg of total RNA were loaded in each lane. **(B)** Abundance of the *mVenus-HA* transcript in the PpsaA:atpA and PatpA:psaA transplastomic strains incubated at 25 and 40°C, respectively, was determined by northern blotting:10 μg of total RNA were loaded in each lane. **(C)** Abundance of the *mVenus-HA* transcript in the PpsaA:psaA, PpsaA:m1, PpsaA:m2, PpsaA:m3 and PpsaA:m4 strains incubated at the indicated temperatures was determined by northern blotting: 4 μg of total RNA were loaded in each lane. In all the experiments, the methylene blue-stained blot is shown as a control for equal loading. The expected size of the *mVenus-HA* transcript with the *atpA* 5′ UTR is 1.1 kb, the size of the *mVenus-HA* transcript with the *psaA* 5′UTR is 0.9 kb. The experiment was performed four times with similar results. The full-size blots are shown in Supplementary Figures S7 and S8.

**Figure 6.**
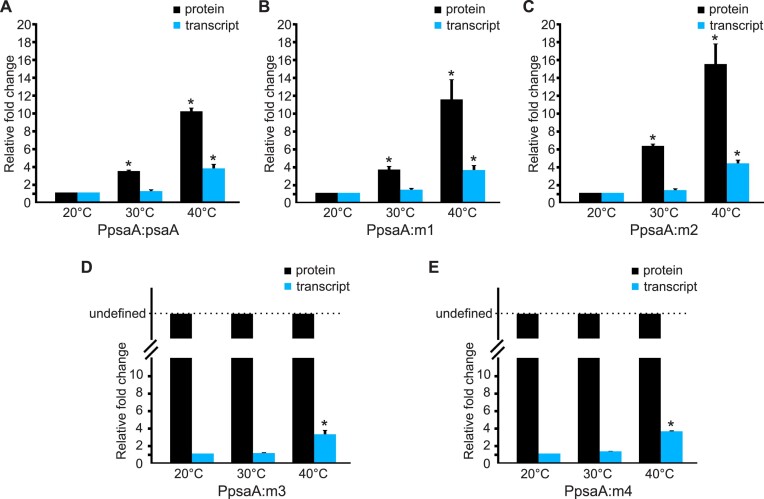
Relative changes in mVenus-HA protein and transcript levels of different transplastomic strains incubated at 20, 30 and 40°C for 6 hours. **(A–C)** The relative fold change of mVenus-HA protein abundance (black bars) across the tested temperatures was determined by western blotting, followed by measurement of band intensities using the Fiji software. The mVenus-HA protein band intensity measured at 20°C was set to 1.0 (see Supplementary Figure S9). **(D,E)** In the m3 and m4 strains, no mVenus-HA protein was detected at 20°C. The relative fold change of mVenus-HA protein abundance in the m3 and m4 strains is, therefore, mathematically undefined (division by zero, represented by broken black bars reaching the horizontal dashed line). **(A–E)** The abundance of *mVenus-HA* transcripts was determined by northern blotting, followed by measurement of band intensities with the Fiji software. The *mVenus-HA* transcript intensity measured at 20°C was set to 1.0. Data are shown as means with error bars representing the standard deviation of three replicates (*n* = 3). Asterisks indicate significant differences (*P* < 0.05) with respect to the corresponding 20°C sample (Welch's *t*-test).

Having demonstrated the temperature responsiveness of the various thermometer constructs at the protein level, we next determined the abundance of the *mVenus-HA* transcripts at different growth temperatures by performing northern blot experiments (Figure 5 and Supplementary Figures S7 and S8). At 40°C, an increase in *mVenus-HA* transcript accumulation was observed in the transformants harbouring the *psaA* 5′ UTR, but not in those containing the *atpA* 5′ UTR (despite the presence of the same promoter in the constructs; c.f. Figures 1A and 2A, and Figure 5A,B). To further investigate the temperature effect on transcript abundance, we compared the strain harbouring the native PpsaA:psaA construct with the strains containing the mutant variants m1, m2, m3 and m4 at 20, 30 and 40°C. In all strains, no substantial accumulation of *mVenus-HA* transcripts was observed upon the temperature shift from 20 to 30°C, while higher *mVenus-HA* mRNA levels were observed at 40°C (Figure 5C). As the accumulation of *mVenus-HA* transcripts at 40°C potentially contributes to the temperature-dependent expression of the reporter protein, we next wanted to disentangle the effects of transcript abundance and thermometer properties on mVenus-HA expression. To this end, we quantified the relative fold changes in transcript levels and protein levels for all variants at the different temperatures. Importantly, the accumulation of the *mVenus-HA* transcripts was comparable at 20 and 30°C (Figure 6), despite a >3-fold increase in protein level seen at 30°C in the psaA, m1 and m2 strains (Figure 6A–C and Supplementary Figure S9). These data support the conclusion that the RNA thermometer acts post-transcriptionally and that the increased accumulation of the mVenus-HA protein at 30°C is not caused by increased transcript amounts. At 40°C, both the elevated transcript levels and the temperature responsiveness of translation may contribute to the increased expression of the reporter protein (Figure 6A–C). Notably, despite the high abundance of *mVenus-HA* transcripts in the m4 strain (Figure 5C), no accumulation of mVenus-HA protein occurred at 40°C (Figure 4J), suggesting that the non-meltable thermometer structure does not permit translation.

### PsaA protein accumulation is independent of the growth temperature

Having shown that the *psaA* 5′ UTR harbours an RNA thermometer that confers temperature-responsive gene expression in both plastids and bacteria, we wanted to determine the possible impact of the thermometer on PsaA protein accumulation in *Chlamydomonas* chloroplasts. Together with PsaB, the PsaA protein forms the photosystem I reaction centre. Photosystem I is a large thylakoid membrane-embedded complex composed of numerous protein subunits and co-factors (including, e.g. light-absorbing pigments and iron-sulfur clusters). The biogenesis of photosystem I is highly regulated (54,55) and depends on the timely availability of all subunits and co-factors (56,57). It is well established that supernumerary subunits are rapidly degraded, and reduced availability of any essential protein subunit will limit photosystem I accumulation (58). In addition, unassembled PsaA has been shown to repress its own translation (56). In the light of all these considerations, it seemed highly unlikely that temperature responsiveness of the *psaA* 5′ UTR would lead to increased accumulation of PsaA protein and/or photosystem I complexes in chloroplasts. Nonetheless, we comparatively analysed both *psaA* mRNA accumulation and PsaA protein abundance at 25 and 40°C (Supplementary Figure S10). As expected, *psaA* transcript amounts did not change significantly with temperature (Supplementary Figure S10A). PsaA protein amounts also did not increase with temperature (Supplementary Figure S10B), in line with the tight autoregulation of PsaA synthesis (56), and the dependence of PsaA accumulation on assembly into photosystem I complexes (57–59).

In conclusion, there is no evidence for the RNA thermometer causing enhanced PsaA protein abundance in response to temperature increases. In fact, the translation efficiency of *psaA* was reported to be reduced upon a short-term (1 h) shift from 25 to 40°C, but showed a recovery trend during prolonged (24 h) heat treatment (60).

### The *psaA* thermometer can boost recombinant protein expression in response to a temperature shift

The *psaA* promoter and 5′ UTR are frequently used to drive recombinant protein expression from the *Chlamydomonas* chloroplast genome (61–64). *Chlamydomonas* is considered mesophilic, and the alga is typically cultured at growth temperatures between 20 and 30°C. The discovery of an RNA thermometer in the *psaA* 5′ UTR opens the attractive possibility to boost recombinant protein accumulation by simply shifting the growth temperature to 40°C prior to harvest of the algal cells.

**Figure 7.**
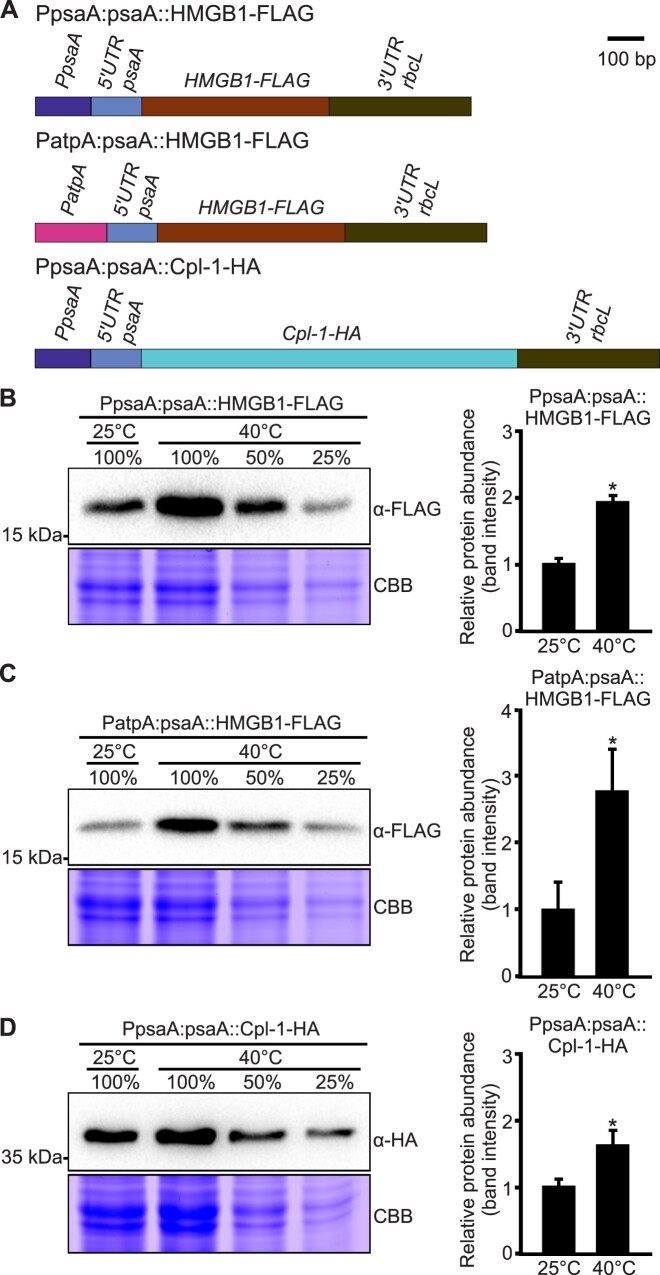
Temperature-induced boosting of pharmaceutical protein expression in *Chlamydomonas* chloroplasts. **(A)** Maps of expression cassettes for HMGB1-FLAG and Cpl-1-HA. Transgene expression is driven by the *psaA* 5′ UTR (harbouring the RNA thermometer) and the *psaA* promoter (PpsaA:psaA) or the *atpA* promoter (PatpA:psaA). **(B–D)** Recombinant protein accumulation in dependence on the growth temperature. Total protein was extracted from cultures incubated at 25°C or shifted to 40°C for 6 h. Immunoblot detection of the therapeutic proteins was performed with the indicated antibodies. The 2-fold dilution series of the 40°C samples were loaded to provide a semiquantitative assessment of protein abundance. Samples of 50 μg protein were loaded as 100%. Coomassie Brilliant Blue (CBB) staining of the protein gel was performed to provide a loading control. Relative protein abundance was quantified by measurement of band intensities. The average band intensity of the samples grown at 25°C (from three independent experiments) was normalized and set as 1.0. Data are shown as means with error bars representing the standard deviation (*n* = 3). Asterisks indicate significant differences (*P* < 0.05) with respect to the 25°C samples (Welch's *t*-test).

To examine whether this provides a generally applicable strategy to increase the yields of recombinant proteins, we tested three pharmaceutical proteins that have been successfully expressed in chloroplasts: (i) the high mobility group protein B1 (HMGB1), which promotes wound healing by inducing cell proliferation and cell migration (46), (ii) the phage-derived endolysin Cpl-1, an antibacterial agent against *Streptococcus pneumoniae*, the causative agent of pneumonia (65,66) and (iii) the p24-Nef fusion protein that combines two HIV antigens that are considered essential components of future AIDS vaccines (40,67). Synthetic codon-optimized version of the three transgenes were inserted into chloroplast expression cassettes that place the coding regions under the control of the *psaA* 5′ UTR in combination with the *psaA* or *atpA* promoter (Figure 7A and Supplementary Figure S11A). To facilitate immunochemical detection of HMGB1 and Cpl-1, for which no antibodies were available, the proteins were epitope tagged with a FLAG-tag and an HA-tag, respectively (Figure 7A).

Transplastomic strains were generated for all three therapeutic proteins and purified to homoplasmy. Algal cultures were then grown at 25°C and either kept at 25°C or shifted to 40°C for 6 hours. The temperature shift resulted in a pronounced increase in recombinant protein expression for HMGB1 (Figure 7B,C). As expected, the yield increase was independent of the promoter, and combination of the *psaA* thermometer with the *psaA* promoter or the *atpA* promoter conferred similar increases in HMGB1 accumulation at 40°C (Figure 7B,C).

Surprisingly, while HMGB1 clearly showed elevated recombinant protein yields at 40°C (and Cpl-1 showed a modest increase in accumulation; Figure 7D), the p24-Nef protein showed decreased accumulation after the temperature shift (Supplementary Figure S11B). In view of the simple melting mechanism underlying RNA thermometer function, we reasoned that this finding is unlikely explained by disturbed thermometer function upon combination with the *p24-nef* coding region. Instead, we suspected that the reduced protein accumulation at 40°C has a post-translational cause, in that the fusion protein is less stable at 40°C. To test this assumption, we constitutively expressed the p24-Nef protein in *E. coli* and compared protein accumulation levels at 25 and 40°C. These experiments revealed that protein accumulation at 40°C was substantially lower than at 25°C (Supplementary Figure S12). As protein synthesis in *E. coli* proceeds with much higher rates at 40°C than at 25°C (68), the decreased p24-Nef accumulation at 40°C must have a post-translational cause and, most likely, is due to reduced protein stability.

Taken together, the temperature-induced enhancement of the expression of the reporter protein mVenus (Figures 1, 2 and 4) and the pharmaceutical proteins HMGB1 and Cpl-1 (Figure 7) demonstrate that the *psaA* thermometer can mediate temperature-controlled protein expression in chloroplasts and boost the production of recombinant proteins by a simple shift in the growth temperature of the algal culture.

## Discussion

Riboregulators are thought to be absent from organellar genomes. In the course of this work, we have discovered an RNA thermometer in the chloroplast genome of the unicellular green alga *Chlamydomonas reinhardtii*. It shows the typical structural features of minimal-size RNA thermometers (14,12), in that it forms a simple hairpin-type secondary structure that resides within the 5′ UTR and comprises the Shine–Dalgarno sequence (Figure 3B). In this way, the Shine–Dalgarno sequence is sequestered in a double-stranded conformation that makes it inaccessible to the ribosome for translation initiation. The secondary structure can be melted by a simple temperature shift, thus inducing protein synthesis (Figures 1, 2 and 4).

The hallmarks of RNA thermometers are their independence of *trans*-acting factors and their function solely based on physical principles (i.e. the melting of an RNA secondary structure). Multiple lines of evidence support the conclusion that the *psaA* 5′UTR has RNA thermometer properties. First, the observed temperature responsiveness is independent of the promoter and the coding region (Figures 1, 2 and 7). Second, the hairpin structure is necessary and sufficient to confer temperature inducibility of gene expression in *E. coli* (Figure 3). Third, mutations modifying the stability of the hairpin structure alter the temperature response of gene expression in a predictable manner (Figure 4). Quantification of transcript and protein abundances in all variants across a wide temperature range disentangled the effects of transcriptional and translational regulation (Figure 6). The RNA thermometer property is particularly obvious when comparing expression at 20 and 30°C: No significant change in the transcript levels is seen, suggesting that the observed temperature-dependent increase in mVenus-HA accumulation is nearly exclusively due to thermometer-mediated translational regulation (Figure 6A–C). Notably, an increased accumulation of *mVenus-HA* mRNAs in all variants was observed at further elevated temperatures (Figure 5), indicating that, in addition to temperature responsiveness of translation, altered transcript abundance also contributes to the accumulation of the reporter protein at 40°C (Figure 6).

Chloroplast biotechnology suffers from a lack of tools for inducible transgene expression (69). In the absence of endogenous control systems (i.e. systems that are encoded in the plastid genome and would respond to chemical inducers or environmental stimuli), the design of synthetic systems has been pursued. Some progress has been made with the design of synthetic riboswitches (35,36,70), but the identification of suitable switches and inducer metabolites has been challenging (34). This is mainly due to (i) the strong dependence of chloroplast gene expression on post-transcriptional (mainly translational) control, and (ii) the presence of large and diverse metabolite pools inside plastids, which make regulation by external metabolite application difficult and cause substantial leakiness of the switches (34). The attraction of RNA thermometers lies in their function as RNA-only translational control elements, and thus, their independence of metabolites or proteins. The *psaA* RNA thermometer and its variants described here potentially provide superior control elements for inducible chloroplast gene and transgene expression. In particular, the engineered variant m3 that shows undetectably low expression at 25°C (the normal growth temperature of *Chlamydomonas* in the laboratory) and substantial induction at 40°C (Figure 4I) is expected to become a useful tool for the regulated expression of endogenous chloroplast genes and the inducible expression of chloroplast transgenes in biotechnology. However, it should be noted that prolonged incubation (i.e. 24 h) of algal cultures under acute high temperature stress (40°C) is not recommended, as the applied heat stress reduces photosynthetic efficiency and inhibits cell growth (71).

The *psaA* promoter and 5′ UTR are widely used to drive transgene expression in *Chlamydomonas* chloroplasts (61–64). Since the yield of recombinant protein is key to the cost effectiveness of any production system in biotechnology (72,73), the possibility to boost protein expression levels by applying a temperature shift before harvest of the algal culture provides a simple and inexpensive method to improve product yields in molecular farming. Also, it will be interesting to test the *psaA* thermometer from *Chlamydomonas* in higher plant plastids to determine whether it also confers heat inducibility to gene and transgene expression in seed plants.

Whether RNA thermometers are more widespread in 5′ UTRs of chloroplast genes in algae and plants is currently unknown and should be investigated in future research. The *psaA* 5′ UTR of algae and land plants are diversified (74). Among Embryophyta, conserved regions in the *psaA* 5′UTR were identified (74). Intriguingly, a Shine–Dalgarno sequence is present upstream of the *psaA* translation start site in the examined species (74). In cyanobacteria (*Synechococcus sp. PCC 7002*), an RNA secondary structure similar to the *Chlamydomonas psaA* thermometer is predicted by the Mfold web server upstream of the *psaA* translation start site. Interestingly, both photosynthetic activity and growth rate of *Synechococcus* increase with temperature (75). Proteomic studies have indicated an increase in PsaA protein abundance at higher temperatures (75). These findings suggest a potential role of the *psaA* thermometer in cyanobacteria. However, more detailed experimental studies are required to test for *psaA* thermometer activity in cyanobacteria. In our present study, we did not observe an effect of the RNA thermometer on PsaA protein abundance in response to increased temperature. In a recent ribosome profiling study, a reduced translational output of *psaA* upon heat stress has been observed in *Nicotiana tabacum* (60). It is noteworthy in this context that, during evolution, the complexity of the regulation involved in the biogenesis of photosystem I has increased substantially (57). For instance, negative feedback loops (control by epistasy of synthesis, CES) regulating the assembly of the photosynthetic complexes have been identified in *Chlamydomonas*, but not in cyanobacteria (56). Given these intricate layers of regulation in algae and land plants, we speculate that the influence of the RNA thermometer on the regulation of photosystem I biogenesis may have diminished during evolution.

In summary, our work reported here has identified a riboregulator in an organellar genome that can be utilized as a convenient tool to facilitate inducible transgene expression and enhance recombinant protein production in chloroplasts by applying a simple shift in the growth temperature. Remarkably, a recent study identified RNA thermometers that regulate nuclear gene expression in *Arabidopsis thaliana* (76). At elevated temperatures, the translation of *PHYTOCHROME-INTERACTING FACTOR 7* (*PIF7*), *HEAT SHOCK FACTOR A2* (*HSFA2*) and *WRKY22* was enhanced. The expression levels of these transcription factors control plant thermomorphogenesis, heat acclimation and other stress responses (76,77). The discovery of RNA thermometers in photosynthetic eukaryotes opens up new research directions in thermoregulation and new biotechnological applications in plants and algae.

## Supplementary Material

gkad816_Supplemental_File

## Data Availability

The data underlying this article are available in the article and in its online supplementary material.

## Notes

Present address: Mengting Wu, Anhui Provincial Key Laboratory of Microbial Control, Anhui Agricultural University, Hefei 230036, China.
