# Supplementary material for: An RNA thermometer in the chloroplast genome of Chlamydomonas facilitates temperature-controlled gene expression
Source: Nucleic Acids Res. 2023 Oct 19;51(20):11386–400. doi: 10.1093/nar/gkad816 (PMC10639063; doi:10.1093/nar/gkad816)
Supplement: gkad816_Supplemental_File [file gkad816_supplemental_file.pdf]

SUPPLEMENTARY DATA for

An RNA thermometer in the chloroplast genome of *Chlamydomonas* facilitates temperature-controlled gene expression

Kin Pan Chung[1], F. Vanessa Loiacono[1], Juliane Neupert[1], Mengting Wu[1], and Ralph Bock[1,*]

[1] Max-Planck-Institut für Molekulare Pflanzenphysiologie, Am Mühlenberg 1, D-14476 Potsdam-Golm, Germany

* To whom correspondence should be addressed. Tel: +49 (0)331 567-8700; Email: rbock@mpimp-golm.mpg.de

Present Address: Mengting Wu, Anhui Provincial Key Laboratory of Microbial Control, Anhui Agricultural University, Hefei 230036, China

This document includes:

Supplementary Figures S1 – S12

Supplementary Table S1

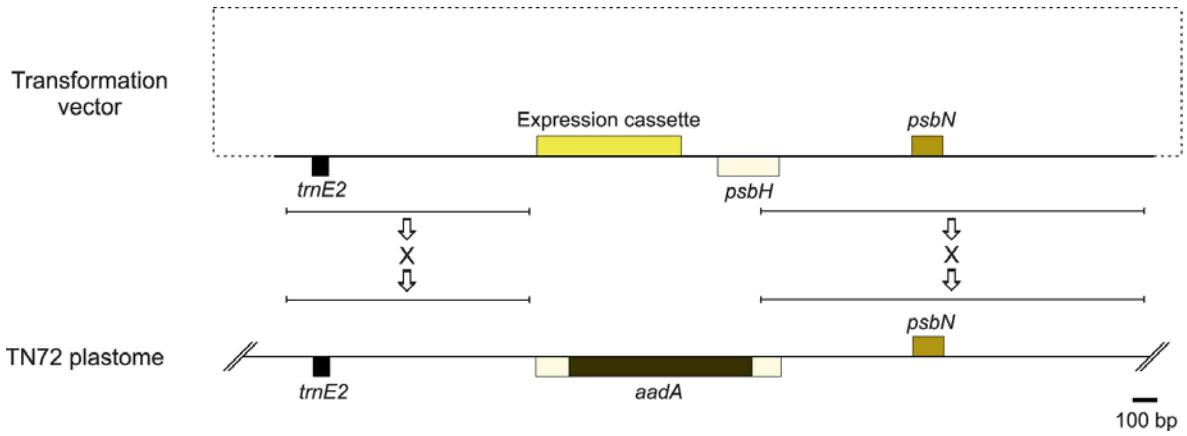

Supplementary Figure S1. Chloroplast transformation strategy using the TN72 mutant strain. Strain TN72 is non-photosynthetic due to disruption of the chloroplast *psbH* gene by insertion of an *aadA* cassette conferring spectinomycin resistance. The strain serves as recipient for chloroplast transformation with vectors that restore a functional *psbH* locus, and thus, photoautotrophic growth. The reporter gene expression cassette is linked to the *psbH* gene, and flanking regions derived from the upstream *trnE2* region and the downstream *psbN* region facilitate transgene integration into the TN72 plastid genome by homologous recombination (indicated by crosses and vertical arrows; flanking homologous regions denoted by horizontal bars).

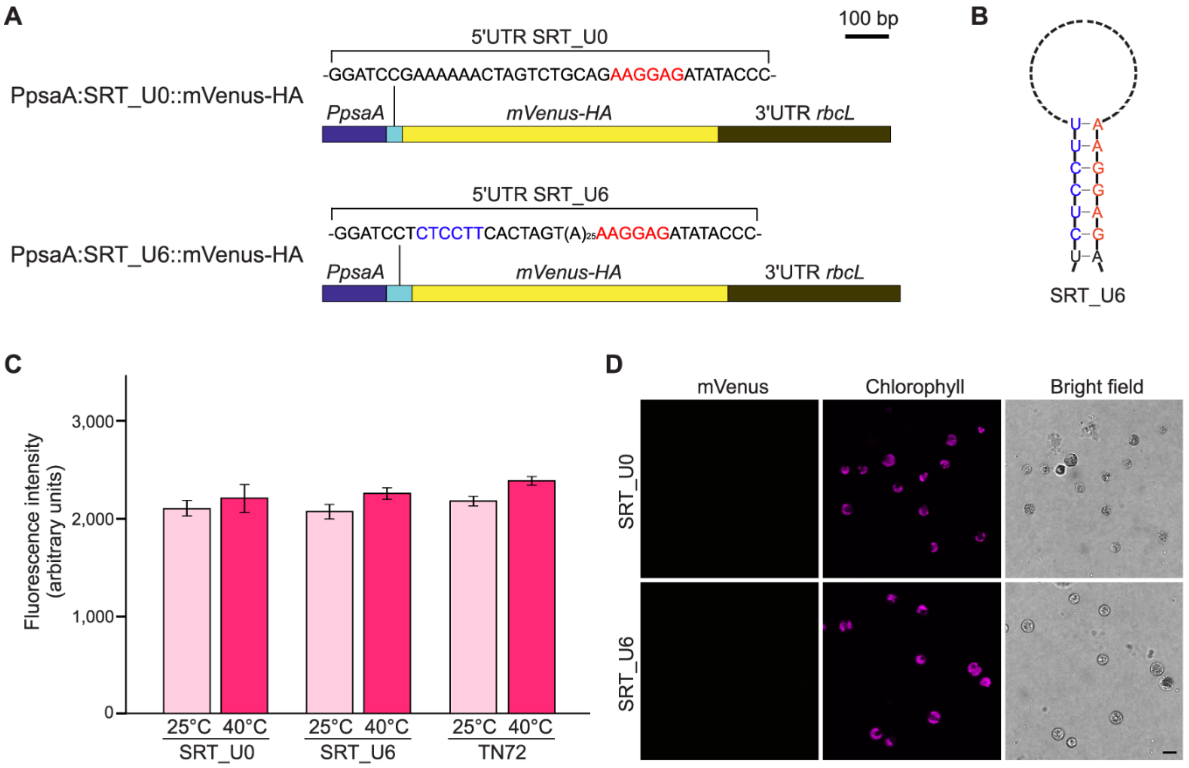

Supplementary Figure S2. Test of synthetic RNA thermometers for function in *Chlamydomonas reinhardtii* chloroplasts. **(A)** Maps of mVenus-HA expression cassettes driven by the plastid *psaA* promoter and the SRT_U0 5'UTR (construct PpsaA:SRT_U0::mVenus-HA), or the *psaA* promoter and the SRT_U6 5'UTR (construct PpsaA:SRT_U6::mVenus-HA). SRT_U0 is comprised of a sequence lacking an RNA secondary structure and does not show thermoinducibility in *E. coli*. SRT_U6 was shown to confer RNA thermometer activity and functioned particularly well in the physiological temperature range when tested in *E. coli*. The Shine-Dalgarno (SD) sequence is indicated in red, the complementary sequence base pairing with it in SRT_U6 is shown in blue. **(B)** Predicted RNA secondary structure of SRT_U6. The SRT_U6 sequence was designed such that complementary base pairing with the SD sequence results in formation of a hairpin-type secondary structure. The number of dashes in the loop corresponds to the number of inter-nucleotide bonds. **(C)** mVenus fluorescence intensity of cultures incubated at 25°C and 40°C. Fluorescence was measured by a microplate reader, and the fluorescence intensities (arbitrary units) are normalized to the OD_{750} values of the cultures. The recipient strain TN72 was used as a negative control. The fluorescence levels of the SRT_U0, SRT_U6 and TN72 strains are comparable, indicating that the measured fluorescence was largely background fluorescence, and the mVenus reporter protein did not accumulate to significant levels in the transplastomic strains. Data are shown as means with error bars representing the standard deviation of three biological replicates (independent cultures, n = 3). **(D)** Confocal microscopy

analysis to visualize the expression of mVenus-HA in the chloroplast. No mVenus signal was observed, suggesting that the SRT_U0 and SRT_U6 5'UTRs do not permit active reporter protein expression in *Chlamydomonas* chloroplasts. Scale bar, 10 µm.

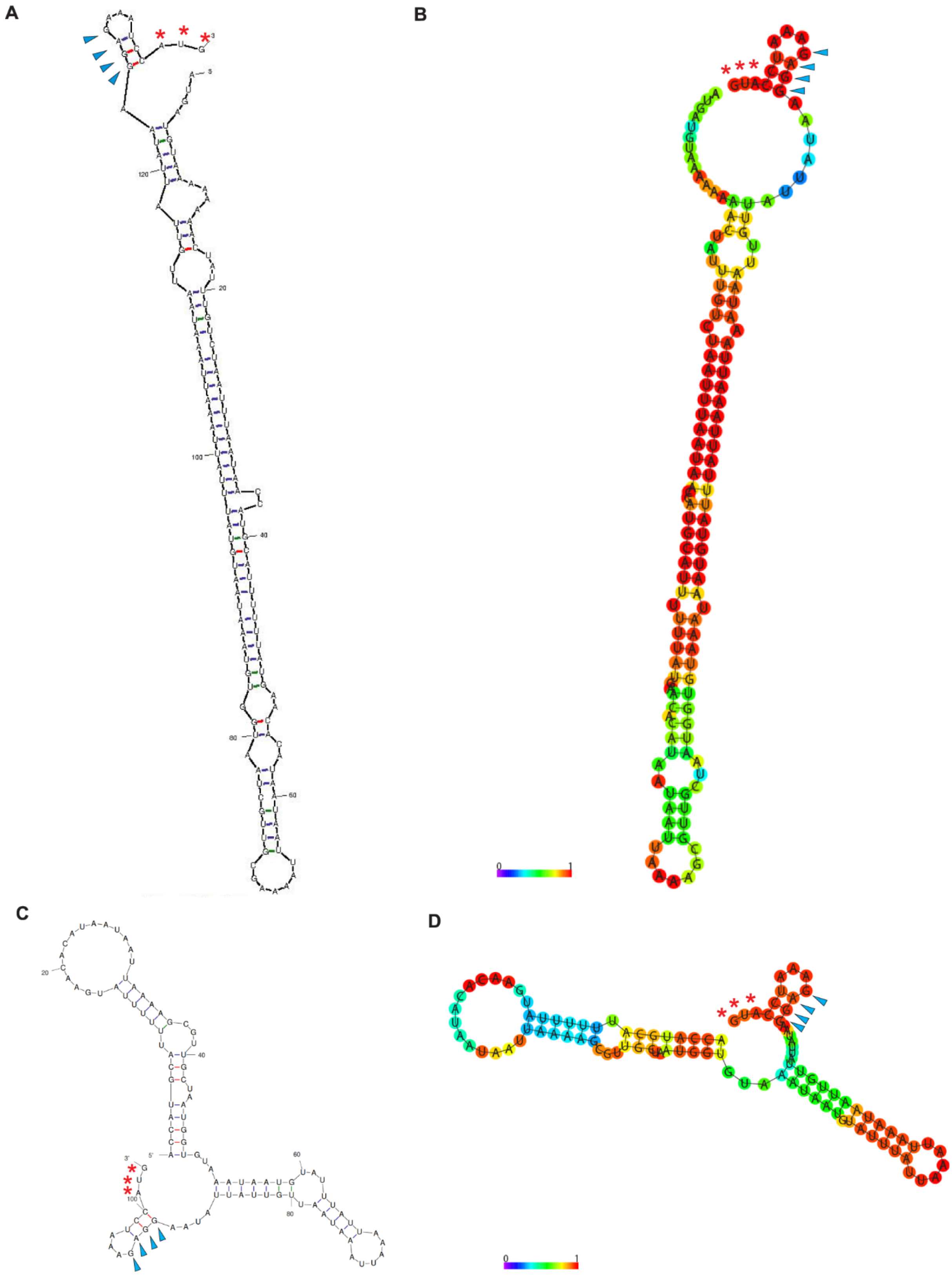

Supplementary Figure S3. RNA secondary structure of the *psaA* 5'UTR as predicted by Mfold 2.3 **(A and C)** or the Vienna RNAfold software **(B and D)**, with the folding temperature set to 25°C. The minimal free energy structures in **(B)** and **(D)** predicted by Vienna RNAfold

are colored by base-pairing probabilities. For unpaired regions, the color denotes the probability of being unpaired. The full-length 5'UTR and the start codon were included in the predictions **(A)** and **(B)**, while only 100 nucleotides of the 5’UTR and the start codon were included in the predictions **(C)** and **(D)**. The translation initiation site (AUG start codon) is marked by red asterisks. Four core nucleotides of the Shine-Dalgarno sequence are indicated by blue arrowheads. The color-gradient heatmap represents the base-pairing probabilities.

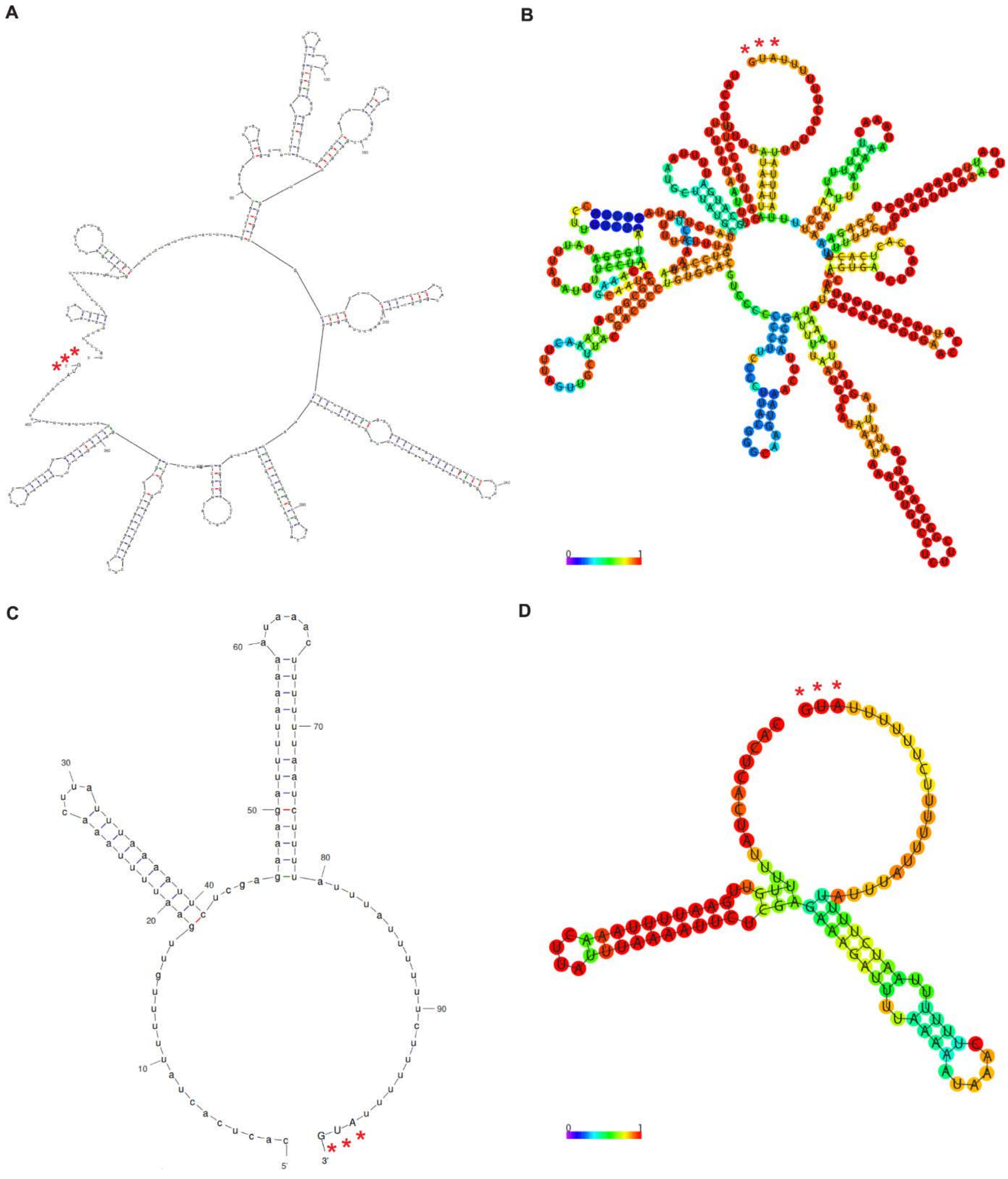

Supplementary Figure S4. RNA secondary structure of the *atpA* 5'UTR as predicted by Mfold 2.3 **(A** and **C)** or the Vienna RNAfold software **(B** and **D)**, with the folding temperature set to 25°C. The minimal free energy structures in **(B)** and **(D)** predicted by Vienna RNAfold are colored by base-pairing probabilities. For unpaired regions the color denotes the probability of being unpaired. The full-length 5'UTR and the start codon were included in the predictions **(A)** and **(B)**, while only 100 nucleotides of the 5’UTR and the start codon were included in the

predictions **(C)** and **(D)**. The translation initiation site (AUG start codon) is marked by red asterisks. The color-gradient heatmap represents the base-pairing probabilities.

A

OD_{750} (PpsaA:psaA::mVenus-HA)				
20°C	25°C	30°C	35°C	40°C
3.64 ± 0.01	3.74 ± 0.01	3.77 ± 0.05	3.90 ± 0.03	3.53 ± 0.03

B

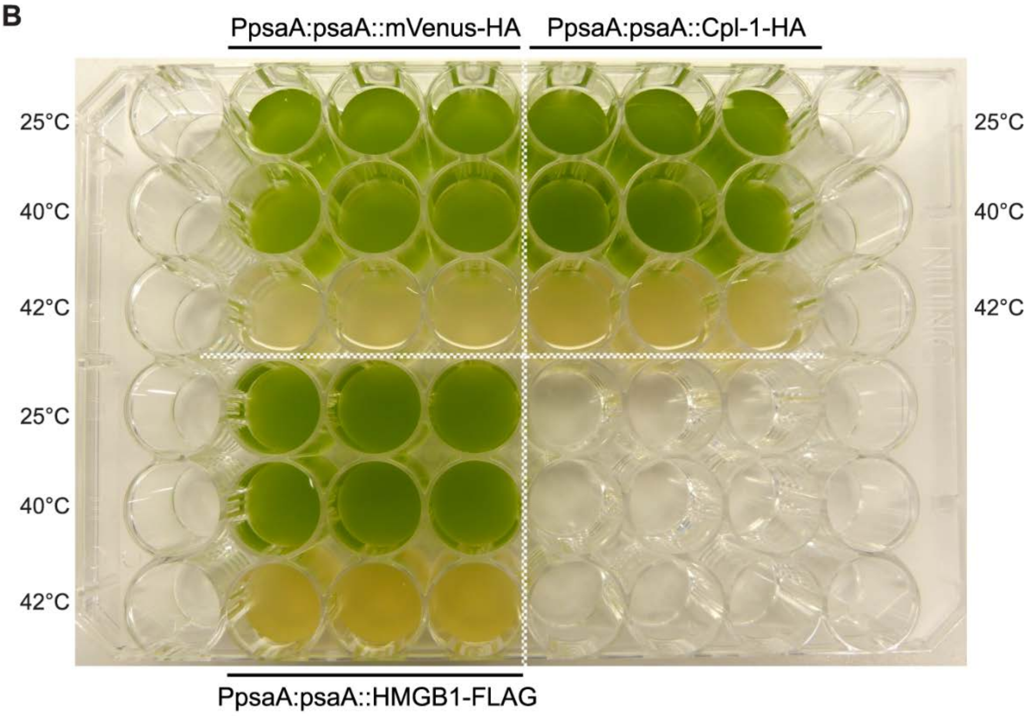

C

	OD_{750}		
	25°C	40°C	42°C
PpsaA:psaA::mVenus-HA	3.72 ± 0.01	3.34 ± 0.01	1.29 ± 0.05
PpsaA:psaA::HMGB1-FLAG	4.51 ± 0.04	4.30 ± 0.02	2.17 ± 0.06
PpsaA:psaA::Cpl-1-HA	3.59 ± 0.03	3.49 ± 0.03	1.59 ± 0.03

Supplementary Figure S5. Heat stress-induced cell bleaching and cell death of transplastomic *Chlamydomonas* strains incubated at 42°C. **(A)** OD_{750} measurement of cultures of the strain (mVenus-HA) incubated at 20°C, 25°C, 30°C, 35°C and 40°C for 6 h. Cell density was determined by measuring the absorbance at the wavelength 750 nm using a microplate reader. The data represent the means ± standard deviation (n = 3). **(B)** Cell cultures of transplastomic strains (mVenus-HA, Cpl-1-HA and HMGB1-FLAG) were incubated at 25°C, 40°C and 42°C for 6 h. Strong bleaching was observed in cultures incubated at 42°C for 6 h. **(C)** OD_{750} measurement of cultures incubated at 25°C, 40°C and 42°C for 6 h. Cell density was determined by measuring the absorbance at the wavelength 750 nm using a microplate reader. Consistent with the bleaching phenomenon, a reduction of the OD_{750} values is observed in cultures incubated at 42°C for 6 h. The data represent the means ± standard deviation (n = 3).

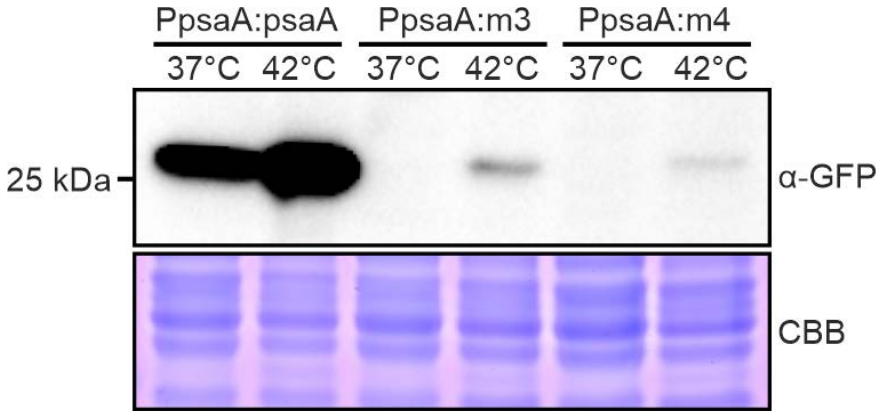

Supplementary Figure S6. Expression of mVenus-HA in *E. coli* transformed with constructs psaA, m3 and m4 at elevated temperature. Total protein was extracted from transformed bacterial cultures incubated at 37°C or 42°C. Immunoblot detection was performed with an anti-GFP antibody (α-GFP). Coomassie Brilliant Blue (CBB) staining of the protein gel was performed to provide a loading control.

A

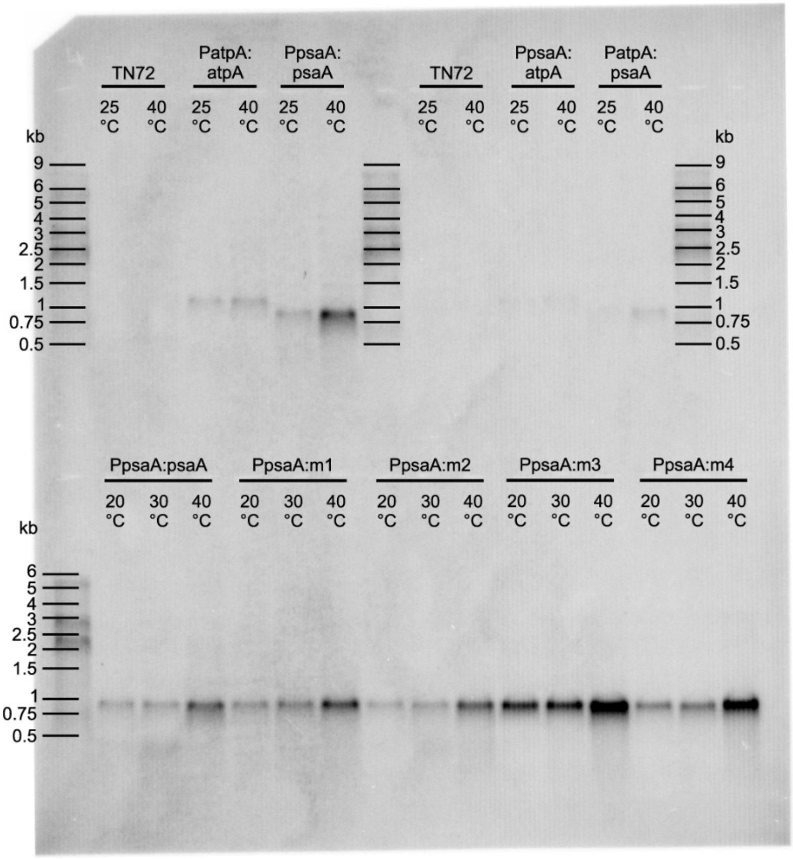

TN72
PatpA: atpA
PpsaA: psaA
TN72
PpsaA: atpA
PatpA: psaA
25 °C
40 °C
kb
9
6
5
4
3
2.5
2
1.5
1
0.75
0.5
PpsaA:psaA
PpsaA:m1
PpsaA:m2
PpsaA:m3
PpsaA:m4
20 °C
30 °C
40 °C

B

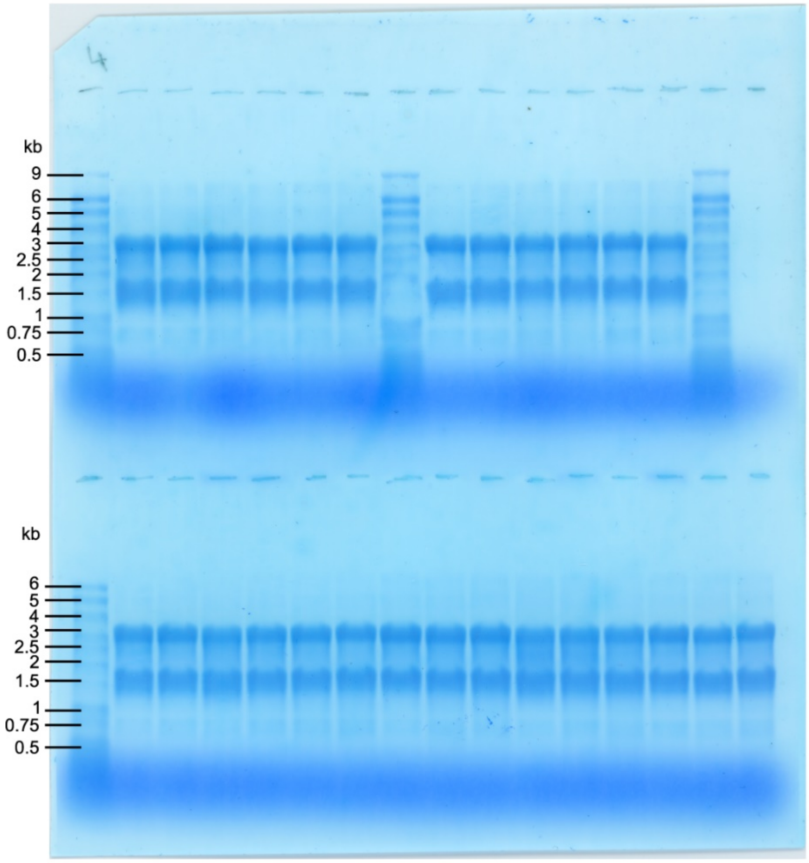

kb
9
6
5
4
3
2.5
2
1.5
1
0.75
0.5

Supplementary Figure S7. Raw data for the expression analysis of the mVenus-HA transgene in transplastomic algal strains by northern blotting (cf. Figure 5A,C). **(A)** Upon heat induction, the abundance of the *mVenus-HA* transcript in the PatpA:atpA, PpsaA:psaA, PpsaA:atpA, PatpA:psaA, PpsaA:m1, PpsaA:m2, PpsaA:m3 and PpsaA:m4 strains incubated at the indicated temperatures was determined by RNA gel blot analyses. The untransformed strain (TN72) was used as negative control. The expected size of the *mVenus-HA* transcript with the *atpA* 5'UTR is 1.1 kb, and the size of the *mVenus-HA* transcript with the *psaA* 5'UTR is 0.9 kb. **(B)** The methylene blue-stained blot is shown as a control for equal loading. 4 µg of total RNA was loaded in each lane. The experiment was performed four times with similar results.

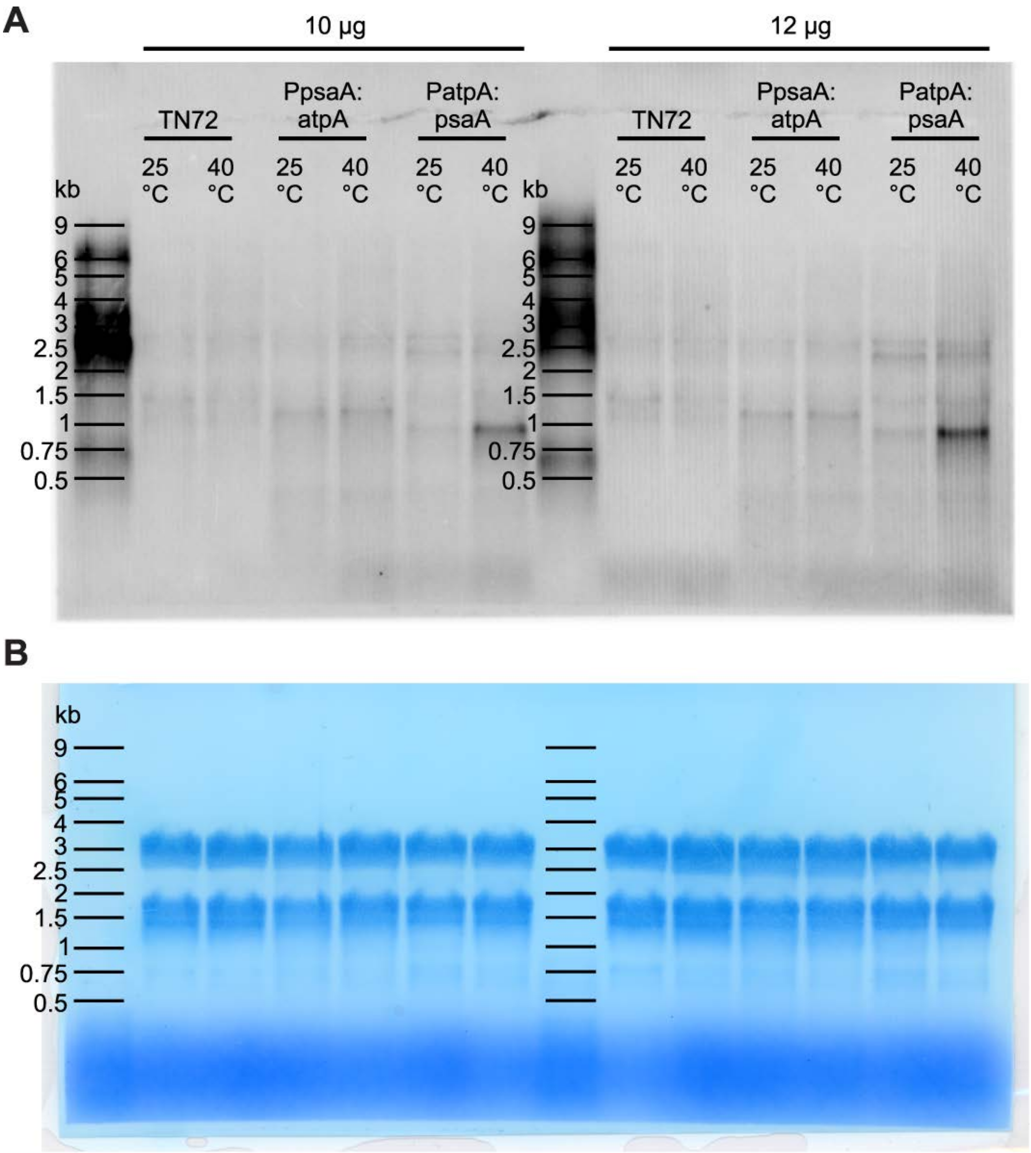

Supplementary Figure S8. Raw data for the expression analysis of the mVenus-HA transgene in transplastomic algal strains by northern blotting (cf. Figure 5B). **(A)** Upon heat induction, the abundance of the mVenus-HA transcript in the PpsaA:atpA and PatpA:psaA strains incubated at the indicated temperatures was determined by RNA gel blot analyses. The untransformed strain (TN72) was used as negative control. The expected size of the *mVenus-HA* transcript with the *atpA* 5'UTR is 1.1 kb, and the expected size of the *mVenus-HA* transcript with the *psaA* 5'UTR is 0.9 kb. **(B)** The methylene blue-stained blot is shown as a control for equal loading. Samples of 10 or 12 µg of total RNA were loaded as indicated above the lanes. The experiment was performed four times with similar results.

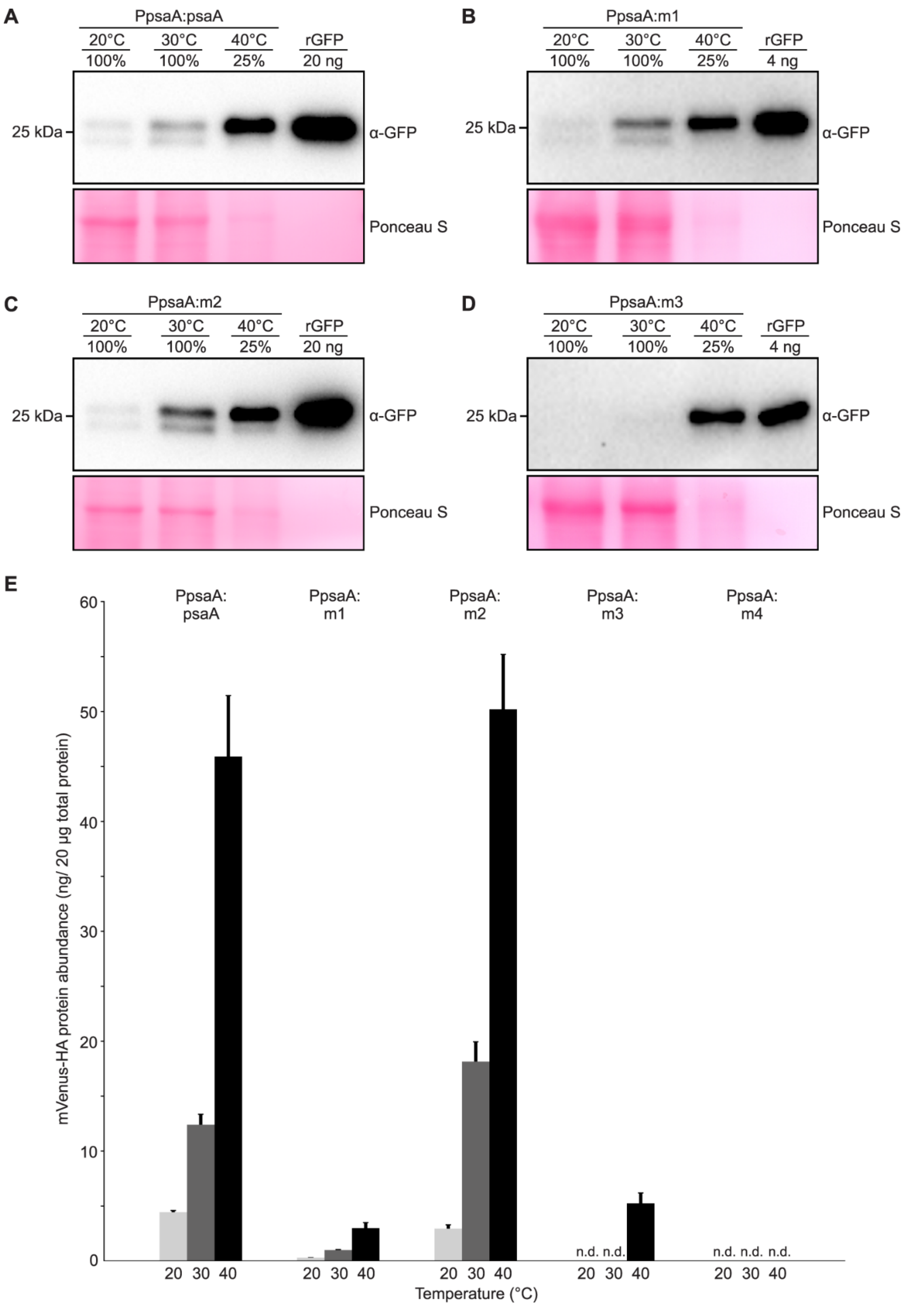
A
PpsaA:psaA
20°C 100%
30°C 100%
40°C 25%
rGFP 20 ng
25 kDa
α-GFP
Ponceau S
B
PpsaA:m1
20°C 100%
30°C 100%
40°C 25%
rGFP 4 ng
25 kDa
α-GFP
Ponceau S
C
PpsaA:m2
20°C 100%
30°C 100%
40°C 25%
rGFP 20 ng
25 kDa
α-GFP
Ponceau S
D
PpsaA:m3
20°C 100%
30°C 100%
40°C 25%
rGFP 4 ng
25 kDa
α-GFP
Ponceau S
E
PpsaA: psaA
PpsaA: m1
PpsaA: m2
PpsaA: m3
PpsaA: m4
mVenus-HA protein abundance (ng/ 20 µg total protein)
0
10
20
30
40
50
60
n.d.
20 30 40
Temperature (°C)

Supplementary Figure S9. Western blotting data for the quantification of relative fold changes in the mVenus-HA protein levels of different transplastomic strains incubated at 20°C, 30°C and 40°C, respectively. **(A–D)** Following the heat induction experiments, total protein was extracted from algal cultures incubated at the indicated temperatures, and accumulation of the reporter protein mVenus-HA was determined with an anti-GFP antibody (α-GFP). Samples of 20 µg protein were loaded as 100% in **(A)** and **(C)**, and samples of 50 µg protein were loaded as 100% in **(B)** and **(D)**. 20 ng **(A** and **C)** and 4 ng **(B** and **D)** of recombinant GFP (rGFP) were used as reference. Ponceau S staining of the blots was performed to provide a loading control. **(E)** Abundance of the mVenus-HA protein (ng / 20 µg of total protein) expressed in different strains. Protein accumulation at the indicated temperatures was determined by western blotting **(A–D)**, followed by band intensity measurement using the Fiji software. In m3 (incubated at 20°C and 30°C) and m4 (incubated at 20°C, 30°C and 40°C), no signal was detected by immunoblotting. The abundance of the mVenus-HA protein of these samples is marked as not detected (n.d.). The experiment was performed three times. Data are shown as means with error bars representing the standard deviation (n = 3).

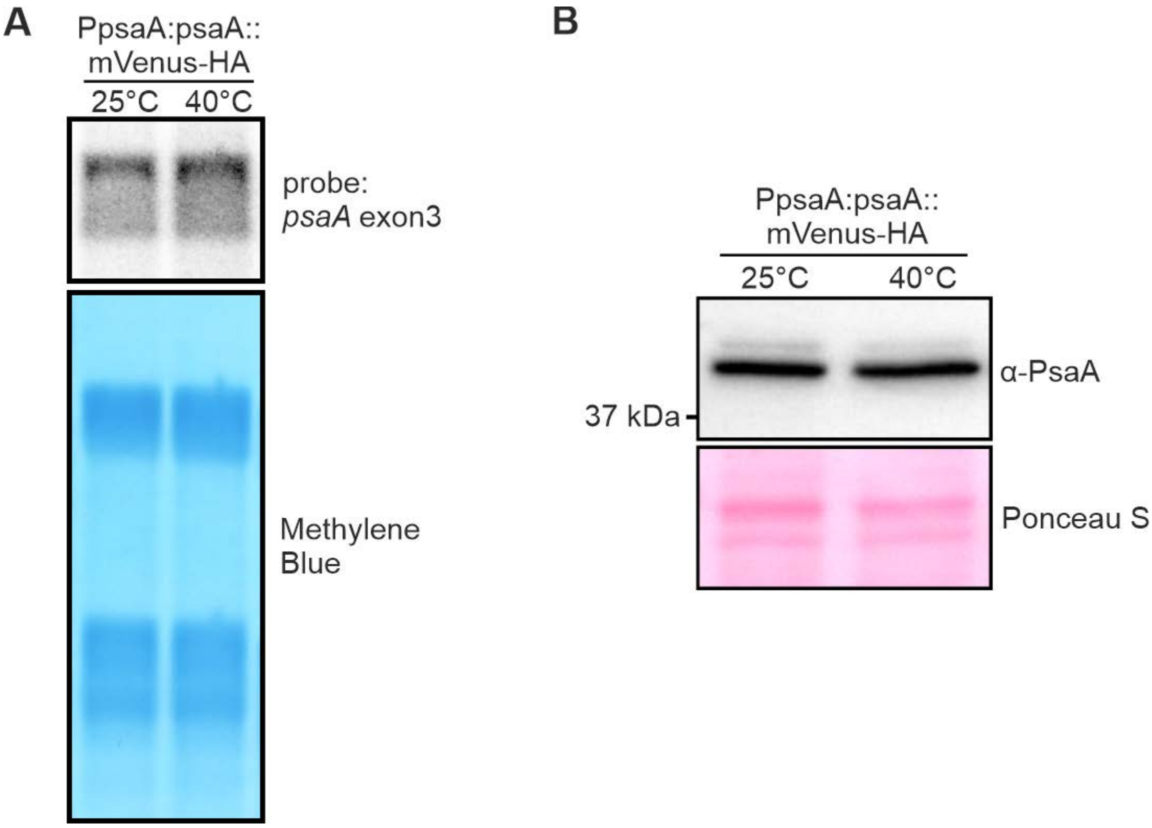

Supplementary Figure S10. Analysis of transcript and protein levels from the endogenous *psaA* locus in the chloroplast genome of *Chlamydomonas*. In the heat induction experiment, the strain PpsaA:psaA::mVenus-HA was incubated at 25°C or 40°C (for 6 h), and *psaA* mRNA accumulation and PsaA protein abundance at the two growth temperatures were compared. **(A)** Analysis of *psaA* mRNA accumulation. Total RNA was extracted from cultures incubated at 25°C or 40°C. Northern blotting was performed to determine *psaA* transcript levels (size of the mature *psaA* mRNA: ~2.7 kb) using a gene-specific probe derived from *psaA* exon 3. Methylene blue staining of the blotted membrane was performed to control for equal RNA loading. **(B)** Analysis of PsaA protein accumulation. Total protein was extracted from cultures incubated at 25°C and 40°C, and immunoblot analysis was performed with an anti-PsaA antibody (α-PsaA). Ponceau S staining served as loading control.

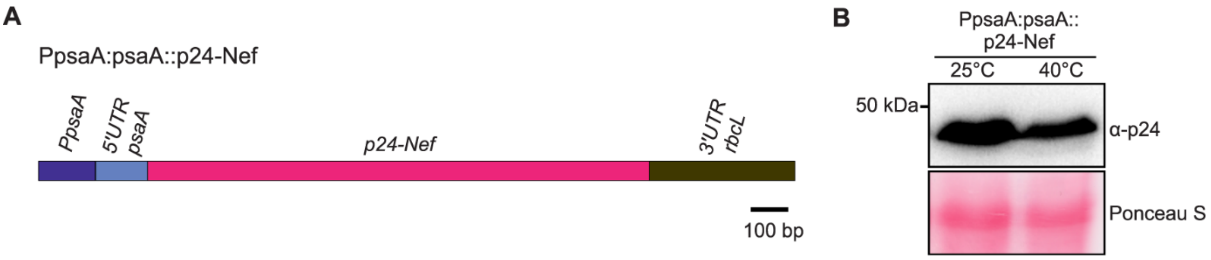

Supplementary Figure S11. Expression of p24-Nef in *Chlamydomonas* chloroplasts. **(A)** Map of the expression cassette for p24-Nef. Transgene expression is driven by the *psaA* 5'UTR (harboring the RNA thermometer) and the *psaA* promoter (PpsaA:psaA). **(B)** Recombinant protein accumulation in a transplastomic strain at different growth temperatures. Total protein was extracted from cultures incubated at 25°C or shifted to 40°C for 6 h. Immunoblot detection of the p24-Nef protein was performed with an anti-p24 antibody (α-p24). Ponceau S staining of the blotted membranes was done to provide a loading control.

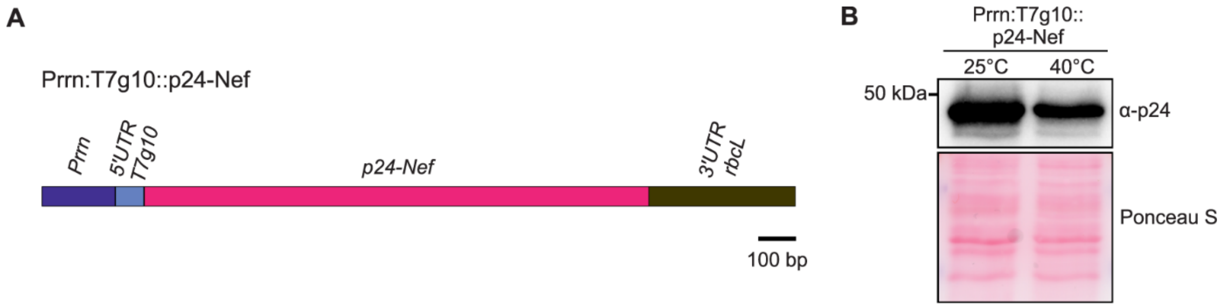

Supplementary Figure S12. Constitutive expression of p24-Nef in *Escherichia coli* confirms reduced protein stability at 40°C. **(A)** Map of the p24-Nef expression cassette driven by the rRNA operon promoter and the 5'UTR from *gene10* of bacteriophage T7 (*T7g10*). **(B)** Comparison of p24-Nef accumulation at 25°C and 40°C. Total protein was extracted from bacterial cultures grown at 25°C and 40°C, and harvested when the OD_{600} had reached 1.0. Immunoblot detection of the p24-Nef protein was performed with an anti-p24 antibody (α-p24). Ponceau S staining provided a loading control.

Supplementary Table S1. List of oligonucleotides used in this study.

**Primer**	**DNA target**	**Sequence 5´→ 3´**
oKPC051	mVenus-HA-F	ATGGTTTCAAAAGGTGAAGAATTATTTACAGG
oKPC052	mVenus-HA-R	TTAAGCATAATCTGGTACATCATATGGATAT
oKPC146	SRT_U0-F	TTATGGTATAATATAACATGGGATCCGAAAAAACTAGTC
oKPC147	SRT_U0-R	CTTGAGTACAAGCTTGCATGTTAAGCATAATCTGGTACATCATATG
oKPC148	SRT_U6-F	TTATGGTATAATATAACATGGGATCCCTCTCCTTCACTAG
oKPC549	PpsaA-F	AAAGAAAAGTGAGCTATTAACGCGTAAGCTTTCTTAATTCAAC
oKPC550	PpsaA-R	AAAAAAAGGTAATGTTATATTATACCATAAATATATTTTTTATTGG
oKPC551	atpA_5UTR-F	AATATAACATTACCTTTTTTTTAATTTGCATGATTTTAATG
oKPC552	atpA_5UTR-R	AATTCTTCACCTTTTGAAACCATAAAAAAGAAAAAATAAATAAAAGATTAAAAAAG
oKPC338	PatpA-F	AAAGAAAAGTGAGCTATTAAACGCGTCTCCAATATAGTAG
oKPC339	PatpA-R	TTTTACATCATAATGTATTTATAAAAAGGTAAATGTATTTATATAGTATTTATATTATAG
oKPC340	psaA_5UTR-F	TAAATACATTATGATGTAAAAAAAACTATTTGTCTAATTTAATAAC
oKPC341	psaA_5UTR-R	CTTGAGTACAAGCTTGCATGCTTAAGCATAATCTGGTACATCATATG
oKPC359	psaA_5'UTR_ΔHP-F	GGGATGCATTTTTTATGAACACATAATAATTAAAAGCGTTGCTAATGGTGTAAATAATGTATTTATTAAATTAAATATTGTTATTATAAGGAGAAAAAAATG
oKPC358	psaA_5'UTR_ΔSD-F	GGGATGCATTTTTTATGAACACATAATAATTAAAAGCGTTGCTAATGGTGTAAATAATGTATTTATTAAATTAAATATTGTTATTATAACCAGAAATGGATG
oKPC301	m1-R	AATTCTTCACCTTTTGAAACCATCCATTTCTGGTTATAATAACAATTATTTAATTTAATAAATAC
oKPC302	m2-R	AATTCTTCACCTTTTGAAACCATAAATTTCTCCTTATAATAACAATTATTTAATTTAATAAATAC
oKPC221	m3-R	AATTCTTCACCTTTTGAAACCATGGAGTTTCCTCCATAATAACAATTATTTAATTTAATAAATAC
oKPC222	m4-R	AATTCTTCACCTTTTGAAACCATGGAGTTTTCACTCCAATAACAATTATTTAATTTAATAAATAC
oKPC439	HMGB1-FLAG-F	TGTTATTATAAGGAGAAATCCATGTCATCATATGCTTTTTTTGTTC
oKPC440	HMGB1-FLAG-R	CTTGAGTACAAGCTTGCATGCTTATGATTTATCATCATCATCTTTATAATC

oKPC356	Cpl-1-HA-F	GGGCCATGGTAAAAAAAAATGATTTATTTGTAGATGTATCTAGTC
oKPC357	Cpl-1-HA-R	GGGGCATGCTTAAGCATAATCTGGAACATCATATGGATAAGCTACAGTAATAAGTCCATCAGGTTCTTTAG
oKPC553	p24-Nef-F	TGTTATTATAAGGAGAAATCCATGGCTAGCGGATCCCC
oKPC554	p24-Nef-R	CTTGAGTACAAGCTTGCATGCCTAGATGGATCCACAATTTTTAAAATATTC
oKPC326	psaA_exon3-F probe	TGATTGTGTTAGCGCTTTGTGC
oKPC327	psaA_exon3-R probe	GCATTTGGTGCTGTTAATTGTGG
oVL528	mVenus-HA-F probe	GTTATGGTTTACAATGTTTTGC
oVL529	mVenus-HA-R probe	TAATACGACTCACTATAGCATAATCTGGTACATCATATGG (T7 promoter sequence indicated in red)
